# Bioclimatic Zoning and Climate Change Impacts on Dairy Cattle in Maranhão, Brazil

**DOI:** 10.3390/ani15111646

**Published:** 2025-06-03

**Authors:** Andressa Carvalho de Sousa, Andreza Maciel de Sousa, Wellington Cruz Corrêa, Jordânio Inácio Marques, Kamila Cunha de Meneses, Héliton Pandorfi, Thieres George Freire da Silva, Jhon Lennon Bezerra da Silva, Marcos Vinícius da Silva, Nítalo André Farias Machado

**Affiliations:** 1 Chapadinha Science Center, Federal University of Maranhão, Chapadinha 65500-000, Brazil; andressa.carvalho1@discente.ufma.br (A.C.d.S.); andreza.maciel@discente.ufma.br (A.M.d.S.); wellington.cruz@discente.ufma.br (W.C.C.); jordanio.marques@ufma.br (J.I.M.); meneses.kamila@ufma.br (K.C.d.M.); mv.silva@ufma.br (M.V.d.S.); 2 Department of Agricultural Engineering, Federal Rural University of Pernambuco, Recife 52171-900, Brazil; hpandorf@hotmail.com (H.P.); thieres.silva@ufrpe.br (T.G.F.d.S.); 3 Cerrado Irrigation Graduate Program, Goiano Federal Institute, Campus Ceres, GO-154, Km 218, Rural Zone, Ceres 76300-000, Brazil; jhon.silva@ifgoiano.edu.br

**Keywords:** biometeorology, GIS, heat stress, geostatistical modeling, temperature–humidity index

## Abstract

To build climate-resilient livestock systems, public policies must be informed by bioclimatic zoning, enabling region-specific interventions and more efficient resource allocation. This study aimed to conduct bioclimatic zoning for dairy cattle farming in the state of Maranhão, Brazil. Big data analysis techniques and predictive geostatistical modeling were applied to historical (2012–2023) and future climate scenarios under intermediate (RCP4.5) and high-intensity (RCP8.5) greenhouse gas emissions. Kriging maps of THI revealed a decreasing north–south thermal gradient, with values exceeding 80 during critical years. Milk yield losses were more pronounced in high-producing cows, reaching up to 5 kg/cow/day under extreme heat. Areas identified as drought-prone exhibited spatial patterns consistent with THI distributions. The projections indicate that, under the RCP 4.5 scenario, over 60% of Maranhão will exhibit average THI values between 78 and 81 by the end of the century. Under the RCP 8.5 scenario, large areas of the state are expected to reach THI values above 86. Under these conditions, estimated milk production losses may exceed 4 kg/cow/day for moderate-yielding animals and 9 kg/cow/day for high-yielding ones, respectively. The results reinforce the importance of bioclimatic zoning to support informed policymaking in the context of climate change.

## Simple Summary

Climate change is increasingly placing Brazilian livestock systems under pressure, threatening livelihoods and food security. Given the urgent need for localized adaptation strategies, this study focused on the state of Maranhão, where scientific assessments remain scarce. We analyzed heat stress risks for dairy cattle based on the THI, using climate data from the past decade and future projections. The analysis of the maps revealed that many areas are already facing dangerous levels of heat stress, with estimated milk losses of up to 5 L per cow per day. These conditions are expected to worsen by the end of the century, with losses reaching up to 9 L in the most vulnerable regions. Moreover, areas traditionally known for strong dairy production and previously considered resilient are projected to become heat stress hotspots, especially in the absence of climate-smart management practices and adequate policy support.

## 1. Introduction

There is broad scientific consensus that the increase in average global surface temperatures constitutes one of the most pressing emerging challenges to sustainable livestock production [1,2,3], especially in tropical countries, where a significant proportion of the global cattle population is concentrated This challenge is particularly concerning due to its deleterious impact on livestock productivity and welfare [4,5,6]. Moreover, the continuous growth of the global population intensifies the demand for livestock products, placing additional pressure on the sector [7].

According to one study [8], the economic losses caused by heat stress in the U.S. livestock industry ranged from USD 897 million to USD 1.77 billion in 2003. Projections outlined by researchers [9] indicate that, by the end of the 21st century, losses in beef and dairy production may reach USD 14.89 billion annually under a low-emission scenario (SSP1-2.6) and up to USD 39.94 billion under a high-emission scenario (SSP5-8.5). Given this significant economic impact, there is a growing need to invest in tools capable of assessing heat load and optimizing thermal stress management in herds [10,11,12].

Dairy cattle, particularly high-yielding breeds, possess physiological traits that render them especially susceptible to heat stress [13]. Their elevated metabolic rate, associated with intensive milk production, leads to increased internal heat generation [5,14,15]. Additionally, these animals have limited sweating capacity and a relatively low surface-area-to-mass ratio, which hampers effective heat dissipation through evaporative and convective means [5,16]. Under conditions of elevated temperature and humidity, cows exhibit physiological responses such as increased respiration rate, elevated rectal temperature, reduced feed intake, and altered blood flow patterns [11,13,16], compromising productivity, reproductive efficiency, and overall welfare.

Bioclimatic indices, particularly the Temperature–Humidity Index (THI), are widely used to monitor thermal stress risk. Although these are indirect indicators based on meteorological parameters [11,17], they enable the establishment of thermal thresholds and classification of animal comfort or discomfort [6,18]. When combined with advanced geostatistical modeling, bioclimatic indices such as the THI enable bioclimatic zoning [19,20,21], allowing for the identification of regions with high productive potential and areas vulnerable to heat stress [20].

Despite Brazil’s significant role in global livestock production, research on the impacts of climate change in the country remains limited. While previous studies have provided valuable information [19,20,21,22], several gaps persist, especially in socioeconomically vulnerable regions [22]. In states like Maranhão, factors such as poverty, limited access to technology, poor logistics infrastructure, and a low adaptive capacity exacerbate the negative effects of heat stress on livestock productivity and welfare [23,24,25,26,27]. These vulnerabilities reduce farmers’ ability to implement mitigation strategies, intensifying production losses and compromising long-term sustainability. Furthermore, the majority of existing research has focused on large-scale climate models and has not sufficiently integrated geospatial analysis or considered region-specific vulnerabilities

In 2024, researchers [22] assessed these impacts on Brazilian livestock using the THI and climate projections for the short (2021–2040), medium (2041–2060), and long term (2061–2080), identifying increased exposure to high heat stress in the South and Southeast, and extreme heat stress in the North, Northeast, and Midwest regions of Brazil. These findings suggest the need for region-specific adaptation measures to mitigate the climatic challenges facing livestock production. Although relevant, the study [22] was based exclusively on a pessimistic high-emission greenhouse gas scenario and did not incorporate a geostatistical analysis through map generation, which may have compromised the spatial accuracy and representativeness of the results [28].

The state of Maranhão, located in northeastern Brazil, is underrepresented in studies on the impacts of climate change, despite its vast territorial extension (331,983 km^2^), comparable to that of several European countries (e.g., Germany, Poland, and Italy), and its strategic position at the intersection of the Amazon, Cerrado, and remaining Caatinga biomes. This results in multiple ecosystems and rich biodiversity. Maranhão holds agricultural significance [29,30], ranking second in grain production within the MATOPIBA agricultural frontier and having a substantial cattle herd of over 10 million heads, with an economic impact exceeding USD 211.08 million annually Additionally, the state hosts an important dairy basin in the southwestern region that supports food security in the Amazon, although the local dairy sector still operates below its potential [31].

In light of the need for regionally tailored adaptive strategies to mitigate climate-related challenges associated with sustainable production, the present study aimed to delineate the bioclimatic zoning for dairy cattle production in the state of Maranhão through the integration of big data analysis techniques and predictive geostatistical modeling. Simultaneously, it sought to prospectively assess the impacts of climate change through temporally stratified projections over short (2011–2040), medium (2041–2070), and long-term (2071–2100) horizons, considering greenhouse gas emission scenarios of intermediate mitigation (RCP4.5) and high intensity (RCP8.5). This approach aims to support sectoral adaptation policies by identifying spatially explicit critical areas, thereby enabling the prioritization of technical interventions and the planning of resilient production systems in the face of projected climate variability.

## 2. Materials and Methods

### 2.1. Characterization of the Study Area

**Figure 1:**
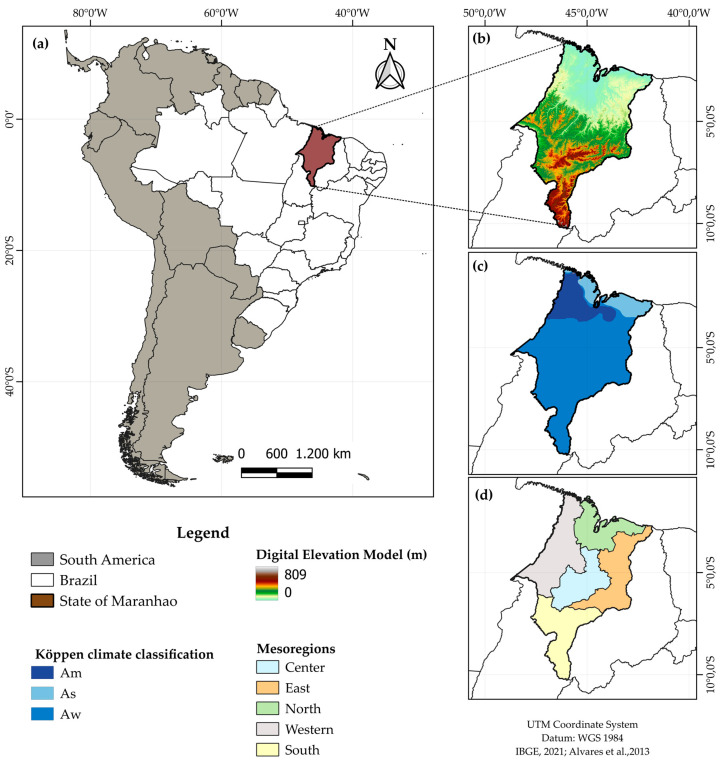
Location of the study area (**a**), digital elevation model (**b**), climate zones according to the Köppen–Geiger classification (**c**), and map of the state of Maranhão divided according to the physiographic zones of its mesoregions (**d**) [35].

The state of Maranhão, the geographic unit analyzed in this study, covers an area of 331,936.949 km^2^ and is located in a biogeographic transition zone between the contrasting climatic regimes of northeastern Brazil. Its geographical position graduates between the drier conditions typical of the semi-arid Northeast and the hyper-humid biomes characteristic of the Northern Region and the Amazon Domain. This ecological interface endows the region with striking environmental heterogeneity, featuring pluviometric, phytophysiognomic, and edaphoclimatic gradients that make it a hotspot for vulnerability and bioclimatic adaptation analyses (Figure 1). Maranhão is divided into five geographic mesoregions: Northern Maranhão, Western Maranhão, Central Maranhão, Eastern Maranhão, and Southern Maranhão [32]. Its hydrography is highly diverse, comprising numerous rivers, lakes, sand dunes, and mangroves, as the state lies within the transition zone between the Cerrado and Amazon biomes [33]. Additionally, in the far eastern part of the state, some municipalities exhibit characteristics of the Caatinga biome [34]. The territorial climate pattern is predominantly classified as tropical with summer rains (Aw), equatorial (Am), and monsoon (As), according to the Köppen–Geiger classification, with an average annual precipitation of 1623 mm and a mean annual temperature of 27.3 °C [35,36].

### 2.2. Data Collection and Processing

**Figure 2:**
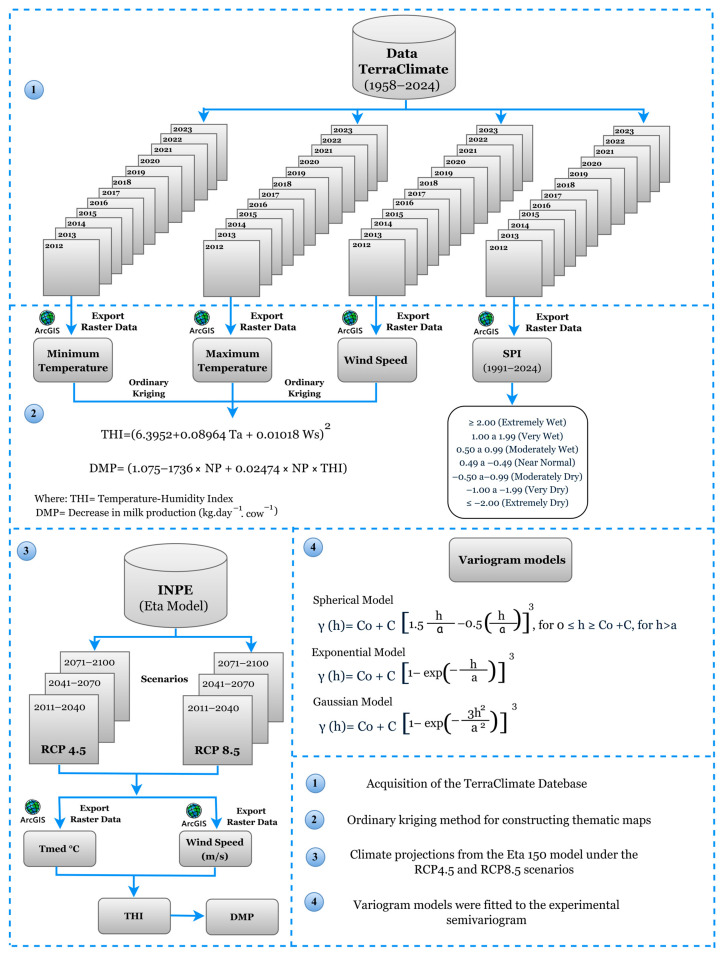
Methodological flowchart of the study, including data collection, interpolation, climate projections, and generation of thematic maps.

A flowchart detailing the methodological procedures can be seen in Figure 2. Minimum temperature (Tmin, °C), maximum temperature (Tmax, °C), and wind speed (Ws, m s^−1^) data were obtained from the TerraClimate database, a freely available global climate dataset [37], with public data accessible at: https://www.climatologylab.org/terraclimate.html (accessed on 20 February 2025). These data were obtained for the historical period from 2012 to 2023, with a spatial resolution of approximately 4 km (1/24°), totaling 20,746 observations (georeferenced sampling points) distributed throughout the state of Maranhão. The choice of TerraClimate was due to its availability of continuous and spatially consistent time series, which is especially relevant in regions like Maranhão, where the meteorological station network has gaps in temporal and spatial coverage [38]. Thus, the use of this dataset ensured a robust basis for regional analyses, even though its relatively coarse spatial resolution may limit the accurate representation of local microclimate characteristics. Nevertheless, its scale and methodological consistency make it suitable for the type of analysis proposed, allowing for the evaluation of climatic patterns.

Subsequently, the mean air temperature (Tair, °C) was calculated, followed by the computation of the annual Temperature–Humidity Index (THI) using Equation (1), as proposed by Silva et al. [39]. Additionally, decrease in milk production (DMP) was estimated using Equation (2) [19,40]. In the present study, DMP was estimated for two standard levels of potential milk production capacity under thermal comfort conditions (PLs) per cow: 10 kg day^−1^ cow^−1^ (PL10) and 25 kg day^−1^ cow^−1^ (PL25). While this approach enables broad regional assessments of heat stress risk, it inherently carries the limitation of not capturing individual or farm-level variability in animal responses to environmental conditions.

(1) $$\mathrm{THI}={\left(6.3952+0.08964\;T\mathrm{air}+0.01018\;\mathrm{Ws}\right)}^{2}$$

(2) DMP=−1.075−1.736×NP+0.02474×NP×THI

where THI is the Temperature–Humidity Index; Tair is the annual mean air temperature (°C); Ws is the annual mean wind speed (m·s^−1^); DMP is the decrease in milk production (kg·day^−1^·cow^−1^); in the present study, NP values of 10 kg day^−1^ cow^−1^ (PL10) and 25 kg day^−1^ cow^−1^ (PL25) were used.

In this study, the interpretation of THI values was guided by thresholds consolidated from the literature, as summarized in Table 1. Additionally, thresholds defined by the Livestock Weather Safety Index (LWSI) were considered, which categorizes heat stress in cattle as follows: normal (THI ≤ 74), alert (74 < THI < 79), danger (79 ≤ THI < 84), and emergency (THI ≥ 84), according to Mader et al. [41]. It is important to highlight that these estimates provide generalized responses to thermal stress. Therefore, they do not explicitly account for differences related to breed, physiological status (e.g., lactation stage, pregnancy), or specific management practices (e.g., access to shade, cooling systems, diet, etc.). Furthermore, the THI model does not incorporate radiative heat load (solar radiation) or behavioral adaptations, such as shade-seeking behavior, which are critical factors influencing the actual thermal stress experienced by animals in field conditions.

We also assessed the impacts of climate change using climate projections from the Eta model, obtained from the platform of the National Institute for Space Research—INPE (available at http://etamodel.cptec.inpe.br/, accessed on 10 February 2025). The Eta model was selected due to its ability to represent, in greater detail, atmospheric phenomena associated with fronts, orographic effects, sea breezes, severe storms, and, more broadly, organized mesoscale systems that are relevant to regional climate dynamics. The operational version used by INPE is a hydrostatic model with a horizontal resolution of 40 km, 38 vertical layers, and a spatial coverage that includes almost all of South America, making it suitable for regional-scale climate studies. For the future projection models under the RCP4.5 and RCP8.5 scenarios, mean temperature (Tmed, °C) and wind speed (Ws, m s^−1^) variables were obtained for the short-term (2011–2040), medium-term (2041–2070), and long-term (2071–2100) periods. These variables were processed using the same methods applied to the historical series (2012–2023), including the calculation of THI and DPM. The RCPs (Representative Concentration Pathways) are greenhouse gas emission scenarios that reflect different radiative forcing trajectories, based on various future emission pathways. The RCP4.5 scenario represents moderate challenges in terms of mitigation and adaptation, along with continued development of fossil fuel resources on a global scale. In contrast, the RCP8.5 scenario indicates significant challenges related to mitigation and suggests a less intensive approach to climate change adaptation [23]. It is worth noting that climate scenarios present uncertainties, mainly due to differences among the models used, which requires caution in interpreting the results as approximate estimates rather than exact predictions [43].

To monitor precipitation anomalies in the state of Maranhão, we used the Standardized Precipitation Index (SPI). The SPI is based on the Gamma probability density function, as shown in Equation (3). The SPI calculation only uses precipitation values [44,45] and is widely applied to analyze drought severity [46]. After calculation, the SPI was analyzed on an annual scale by mesoregions and for the entire state of Maranhão, categorized according to Table 2.

(3) $$F\left(x\right)=\int_{0}^{x}f\left(x\right)\mathrm{dx}=\frac{1}{\Gamma (\alpha )\beta^{\alpha }}\int_{0}^{x}x^{\alpha -1}e^{-\frac{x}{\beta }}\mathrm{dx}$$

where α is the shape parameter (α > 0); β is the scale parameter (β > 0), both determined using the maximum likelihood method; and x is the precipitation amount (a variable dependent on α and β).

### 2.3. Data Analysis

Geostatistical techniques were applied to explore the spatial variability in meteorological data, THI, and DPM. The interpolation for the construction of thematic maps was performed using the ordinary kriging method (Equation (4)). ArcGIS Pro version 3.4, developed by the Environmental Systems Research Institute (ESRI), was used to determine the spatial dependence between pairs of observations through an experimental semivariogram (Equation (5)), based on the assumptions of intrinsic stationarity. Kriging is an advanced geostatistical interpolation method that estimates unknown values at unsampled locations based on the spatial correlation structure of the measured data [48]. Unlike simpler methods, kriging not only considers the distance between points, but also the degree of spatial variability, producing more accurate and reliable estimates [49]. Moreover, kriging provides an estimation of uncertainty associated with the interpolated values, which is particularly important in environmental and climatic studies [20,48,49].

(4) $$\bar{Z}(x_{o})=\sum_{i=1}^{n}\lambda_{i}Z\left(x_{i}\right)$$

where $\bar{Z}(x_{o})$ is the estimated (interpolated) value at location $x_{o}$; $\lambda_{i}$ is the weight assigned to the sampled value $Z\left(x_{i}\right)$ at location ${\;x}_{i}$; $Z\left(x_{i}\right)$ is the sampled value at point $x_{i}$; n is the number of sampling points used for the interpolation; and the sum of the weights $\lambda_{i}$ must be equal to 1.

(5) $$\gamma (h)=\frac{1}{2N(h)}\sum_{i=1}^{N(h)}{\left[Z\left(x_{i}\right)-Z(x_{i}+h)\right]}^{2}$$

where γ(h) is the experimental semivariance estimator, obtained from the sampled values Z(Xi), Z(Xi + h); N(h) is the number of pairs of measured values separated by the lag vector or distance h; h is the distance between sample pairs; and Z(Xi) and Z(Xi + h) are the values of the i-th observation of the regionalized variable, collected at points Xi e Xi + h (i = 1, …, n), separated by the vector h.

The degree of spatial dependence (DSD) in this study was classified as strong when less than 25%, moderate when between 25% and 75%, and weak when greater than 75%, as established by Cambardella et al. [50]. Three variogram models (e.g., spherical, exponential, and Gaussian) were fitted to the experimental semivariogram, as represented by Equations (6), (7), and (8), respectively.

Spherical Model

(6) $$\gamma \left(h\right)=C_{0}+C\;\left[1.5\frac{h}{a}-0.5\;{\left(\frac{h}{a}\right)}^{3}\;\right],\;\mathrm{for}\;0\leq h\geq a\;C_{0}+C,\;\mathrm{for}\;h>a$$

Exponential Model

(7) $$\gamma (h)=C_{0}+C\left[1-\exp\left(-\frac{3h}{a}\right)\right]$$

Gaussian Model

(8) $$\gamma (h)=C_{0}+C\left[1-\exp\left(-\frac{3h^{2}}{a^{2}}\right)\right]$$

where γ(h) is the experimental semi-variance estimator; C_0_ + C is the sill; C_0_ is the nugget effect; C is the variance dispersion; h is the distance between sample pairs; and a is the range (m).

The performance and accuracy of the models were evaluated through cross-validation by comparing the predictions with the measured data. For this purpose, five statistical metrics were used: mean error (ME), mean squared error (MSE), average standardized error (ASE), root mean square error (RMSE), and root mean square standardized error (RMSSE), as defined in Equations (9), (10), (11), (12), and (13), respectively.

(9) $$\mathrm{ME}=\frac{1}{N}\sum_{i=1}^{N}\left[Z\left(x_{i}\right)-Z(x_{i})\right]$$

(10) $$\mathrm{MSE}=\frac{1}{N}\sum_{i=1}^{N}\left(\frac{Z\left(x_{i}\right)-Z(x_{i})}{\sigma_{1}}\right)$$

(11) $$\mathrm{ASE}=\sqrt{\frac{1}{N}\;\sum_{i=1}^{N}\left(\sigma_{1}\right)}$$

(12) $$\mathrm{RMSE}=\sqrt{\frac{1}{N}\;\sum_{i=1}^{N}{\left[\;Z\left(x_{i}\right)-Z(x_{i})\right]}^{2}}$$

(13) $$\mathrm{RMSSE}=\sqrt{\frac{1}{N}\sum_{i=1}^{N}{\left\{\frac{Z\left(x_{i}\right)-Z(x_{i})}{\sigma_{1}}\right\}}^{2}}$$

The data were subjected to descriptive statistical analysis to obtain the mean, extreme values, standard deviation, and coefficient of variation (CV, %), which was categorized as low (CV < 12%), medium (CV = 12–24%), or high (CV > 24%), according to [51]. The normality of the residuals was verified using the Shapiro–Wilk test (*p* < 0.05).

## 3. Results and Discussion

### 3.1. Historical Scenario

Table 3 presents the descriptive statistics of the annual THI values for the years 2012 to 2023. Overall, the annual mean THI values ranged from 78.584 ± 1.579 (2012) to 77.117 ± 1.131 (2022), indicating a relatively homogeneous pattern, with no major interannual variations. The coefficient of variation remained below 24%, indicating low data dispersion [51], further reinforcing the homogeneity pattern observed during the evaluated period. However, the maximum THI values exceeded 79 in all years, reaching the danger threshold defined by the Livestock Weather Safety Index (THI > 79) [41]. Additionally, THI values above 79 indicate a high risk of heat stress for dairy cattle (Table 1). A summary of descriptive statistics for air temperature (Tair, °C) and wind speed (Ws, m s^−1^) can be found in Supplementary Material Tables S1 and S2, respectively. The annual Tair ranged from 26.468 °C (2022) to 27.374 °C (2016), with minimum values between 23.133 °C (2022) and 24.308 °C (2019), and maximum values from 28.141 °C (2022) to 29.416 °C (2016). Regarding Ws, the annual means fluctuated between 1.147 m s^−1^ (2021) and 1.596 m s^−1^ (2015), with minimum values from 0.758 m s^−1^ (2020) to 1.290 m s^−1^ (2017), and maximum values ranging from 1.825 m s^−1^ (2022) to 2.729 m s^−1^ (2012).

**Figure 3:**
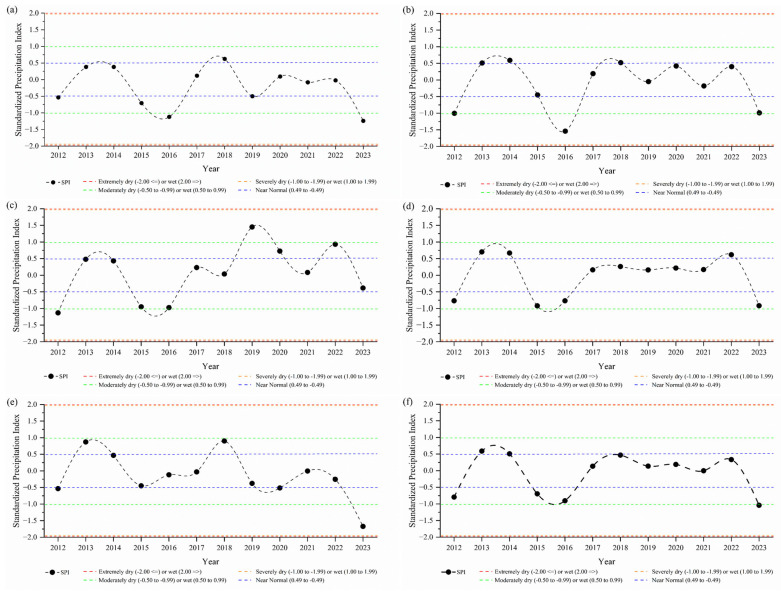
Standardized Precipitation Index (SPI) for the mesoregions of Maranhão Central (**a**), East (**b**), North (**c**), West (**d**), South (**e**), and the entire state (**f**).

The years 2015, 2016, and 2017 were characterized as having the highest continuous average THI values, at 78.263, 78.584, and 78.178, respectively. Apparently, drought was closely associated with heat load and the risk of animal heat stress, and drought events (such as those observed in 2015 and 2016) tended to have persistent effects on THI values in subsequent years. This highlights the need for planning and implementation of public policies aimed at managing livestock production environments, particularly in regions affected by drought events. To understand the effects of droughts across of the state of Maranhão, Figure 3 illustrates the annual Standardized Precipitation Index (SPI-12) from 2012 to 2023 for the mesoregions and the entire state of Maranhão. According to the methodology established by et al. [47], the Central mesoregion (Figure 3a) experienced two drought years—2016 and 2023—while the East mesoregion (Figure 3b) recorded three drought years (2012, 2016, and 2023), both regions being the most affected by “Severe Drought” events. For the other mesoregions, the SPI dynamics ranged from wet conditions to “Moderately Dry” periods (Figure 3). Across the entire state of Maranhão, four drought years were observed—2012, 2015, 2016, and 2023. Of these, 2016 and 2023 were classified as “Severe Drought,” while 2012 was categorized as “Moderate Drought”.

Table 4 presents the statistics used to evaluate the performance of the geostatistical models applied to the THI values, tested individually for the years 2012 and 2023. The RMSSE values indicated the better performance of the spherical model (0.74–0.83), mainly above 0.80. The exponential model presented underestimation (0.58–0.64), while the Gaussian model had intermediate performance (0.65–0.78). The proximity between the RMSE and ASE values was also observed, justifying the choice of the spherical model in the present study to generate the THI kriging maps for Maranhão. A summary of the descriptive statistics for temperature and wind speed can be found in Supplementary Material Tables S1 and S2.

Table 5 presents the main parameters of the adopted geostatistical model, including the nugget effect, sill, and range, as well as the degree of spatial dependence, which is essential for characterizing the variability structure of the data [49]. Information regarding the cross-validation of the geostatistical models tested for annual temperature and wind speed values can be found in Supplementary Material Tables S3 and S4. The range, a fundamental parameter in geostatistics, defines the maximum distance of spatial dependence between samples and is essential for modeling the variability of environmental phenomena [49,52]. In the present study, using data from TerraClimate, the range values varied between 5500 and 9000 m, indicating strong spatial correlation at this scale. Supporting this finding, Cerón et al. [53] compared spatial interpolation methods for annual and seasonal precipitation in two biodiversity hotspots in South America and reported similar ranges. This extent suggests that climatic processes operate on a large scale, corroborating studies that highlighted the influence of atmospheric and topographic patterns on the spatial distribution of meteorological data [54].

**Figure 4:**
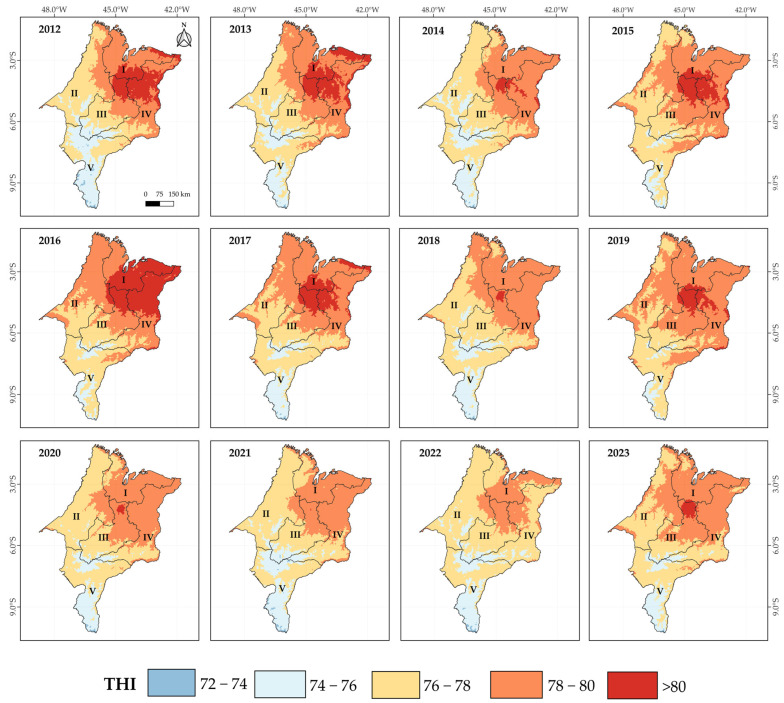
Kriging maps of THI from 2012 to 2023 in the mesoregions of Maranhão: I—North, II—West, III—Central, IV—East, and V—South.

Kriging maps of the spatiotemporal distribution of annual THI in the state of Maranhão are presented in Figure 4. The spatial variability and accuracy of the interpolation models were assessed through experimental semivariograms and cross-validation procedures, as detailed in Figure S1 (Supplementary Material). It can be observed that the Northern, Eastern, and the far north of the Central mesoregions of Maranhão concentrated the highest THI values, with extensive areas in 2012 and 2016 reaching the extreme heat stress level for dairy cattle (Table 1). The Western mesoregion showed moderate heat stress conditions, with THI values generally between 76 and 79, especially during the years 2014 and 2020–2023. However, in years such as 2016–2019, there was an expansion of areas with THI > 80 within this mesoregion. On the other hand, the Southern mesoregion consistently showed the lowest THI values over the evaluated period, with prevailing conditions of moderate heat stress or no significant risk of heat stress, according to Table 1.

Furthermore, as shown in Figure 4, the temporal analysis revealed that the years 2012, 2013, 2016, and 2017 exhibited the largest territorial extents with THI > 80, indicating a higher risk of heat stress and thermal emergency conditions for the animals, according to the Livestock Weather Safety Index (LWSI) [41]. A decreasing trend in the extent of areas with an THI above 80 has been observed since 2020. However, the spatial pattern of higher severity in the Northern and East mesoregions has persisted, highlighting the latitudinal gradient in the state, with greater heat stress risk in the northern and eastern regions, particularly in the northeastern part of Maranhão. Kriging maps of mean temperature, wind speed, all experimental semivariograms, and cross-validation results, including the THI kriging maps, are available in the Supplementary Materials.

The explanation for this result is complex and multifaceted. Firstly, this trend can be partially attributed to the higher altitude of the Southern mesoregion of Maranhão (Figure 1), where temperatures tend to be milder (see Figure S2 in the Supplementary Materials). Additionally, the Central and East mesoregions lie within the rainfall transition zone, bordering the semi-arid region, and exhibit the lowest precipitation indices in the state. However, the primary factor lies in the influence of meteorological systems such as the Intertropical Convergence Zone [55,56,57], combined with the effects of the El Niño–Southern Oscillation (ENSO) phenomenon [53,55], which significantly alter temperature and precipitation patterns, more intensely affecting the Northern mesoregion and, to a lesser extent, the Eastern and Central mesoregions of Maranhão [56].

According to the THI kriging maps for the last decade (Figure 4), it is evident that the Central and East mesoregions experienced the greatest impacts from droughts classified as “Severe Drought”, highlighting the sensitivity of THI dynamics to ENSO phenomena. Supporting the findings of this study, Silva et al. [58], who characterized precipitation patterns and monthly wet–dry periods using the Standardized Precipitation Index (SPI) from 1990 to 2019 along the southern coast of the Brazilian Northeast and employed geostatistical interpolation methods, emphasized that SPI is highly sensitive to rainfall dynamics across multiple time scales. Abrupt changes in precipitation in the data series lead to transitions toward “extreme rainfall” or “extreme drought” events, particularly influenced by ENSO phenomena, especially in the Brazilian Northeast region.

Our analysis revealed that the years 2012, 2013, 2016, and 2017 showed the largest territorial extents with THI > 80, indicating a higher risk of heat stress and thermal emergency conditions for animals according to the LWSI. This period was marked by a significant reduction in rainfall and an increase in average temperatures (see Figure S2 in the Supplementary Materials), severely compromising the region’s climatic balance [55,57,59,60]. Carvalho et al. [55] pointed out that this period corresponded to one of the most severe droughts ever recorded in the northeastern semi-arid region, beginning in 2012 and worsening in 2015, with serious impacts on agricultural and livestock production. The years 2015, 2016, and 2017, in particular, were influenced by an extreme El Niño event, as reported by Marengo et al. [43] and Santos et al. [44], which contributed to the intensification of the observed climate anomalies.

Supporting this perspective, De Sousa et al. [61] highlight that the state of Maranhão experienced successive drought events between 2010 and 2016, strongly influenced by the El Niño phenomenon in 2015, which intensified temperature increases and significantly reduced relative humidity. Additionally, Santos et al. [57] through an analysis of climatic indices, identified an increase in negative precipitation anomalies between 2012 and 2019, with particular emphasis on the periods of 2012–2013 and 2015–2017, which were considered especially critical. Therefore, the relationship between the SPI and THI appears to be strong in the state of Maranhão, as evidenced by the results presented in our study. The occurrence of droughts and the spatial observation of the THI revealed that drought events exacerbate heat stress conditions, especially in areas with lower rainfall frequency, such as the Central and East regions, when considering the entire data series. Orographic conditions combined with higher precipitation levels help mitigate the effects of the THI, characterizing zones with more favorable thermal conditions for dairy cattle production.

When spatializing the SPI across Maranhão (see Figure S8 in the Supplementary Materials), we observed that the most severe droughts consistently overlapped with regions experiencing the highest thermal stress. A notable exception was the southern region of the state, due to complex local meteorological dynamics, as explained below. The meteorological phenomena related to droughts in the Brazilian Northeast are mainly influenced by air masses from the North Atlantic and the warming and cooling of the Equatorial Pacific, as well as air masses originating from the Amazon rainforest [58,62]. When analyzing the most intense drought period in the Brazilian Northeast, from 2012 to 2016, a common pattern of greater drought intensity was observed, especially in the years 2012 and 2015 (see Figure S8 in the Supplementary Materials). In subsequent years, however, anomalies appeared in the southern mesoregion of Maranhão. This region exhibits windward and leeward characteristics and is influenced by an atmospheric damping zone marked by high meteorological heterogeneity.

As a result, droughts or rainfall events can be highly localized, without affecting the entire state. This variability is related to a steep altitudinal gradient, ranging from 0 to 809 m (Figure 1), and a narrow continental strip that is subject to strong influences from North Atlantic air masses. These factors affect the climate at multiple scales, including local processes such as convection, mesoscale phenomena like sea breeze circulation and trade winds, and synoptic systems involving frontal systems and the influence of the Equatorial Continental (mEc), Tropical Atlantic (mTa), and Tropical Continental (mTc) air masses, all of which can intensify or suppress rainfall. In other words, the climate of the southern Maranhão region is subject to the influence of various meteorological systems that induce precipitation extremes, either above or below the historical average. This region, which displays such distinctive behavior, is also the area with the highest topographic elevations, contributing to higher rainfall regimes. It is characterized by an atmospheric damping zone, which may lead to adverse conditions of drought and/or excessive rainfall.

**Figure 5:**
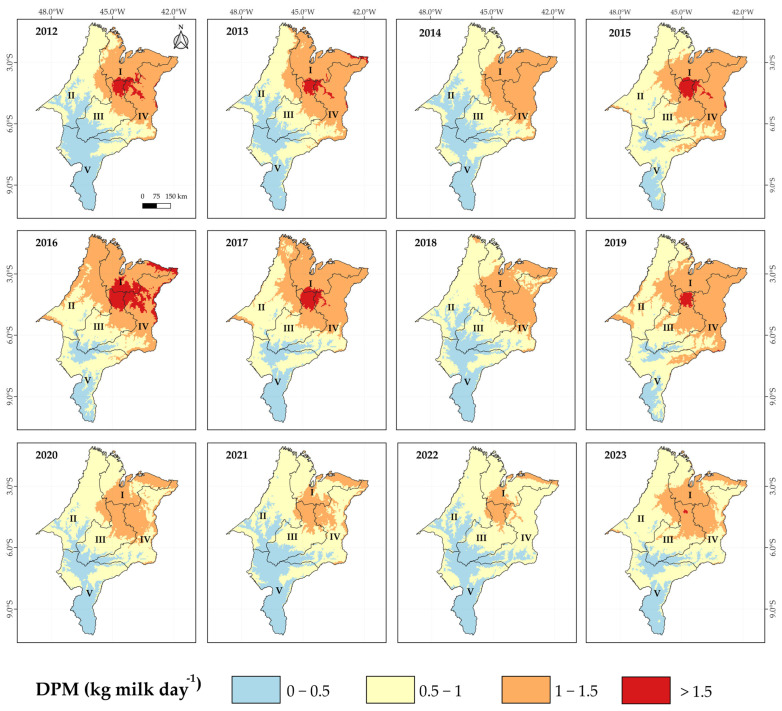
Kriging maps of the decrease in milk production (DMP) relative to the potential production capacity under thermal comfort conditions per cow: 10 kg day^−1^ cow^−1^ (PL10), from 2012 to 2023 in the mesoregions of Maranhão: I—North, II—West, III—Central, IV—East, and V—South.

**Figure 6:**
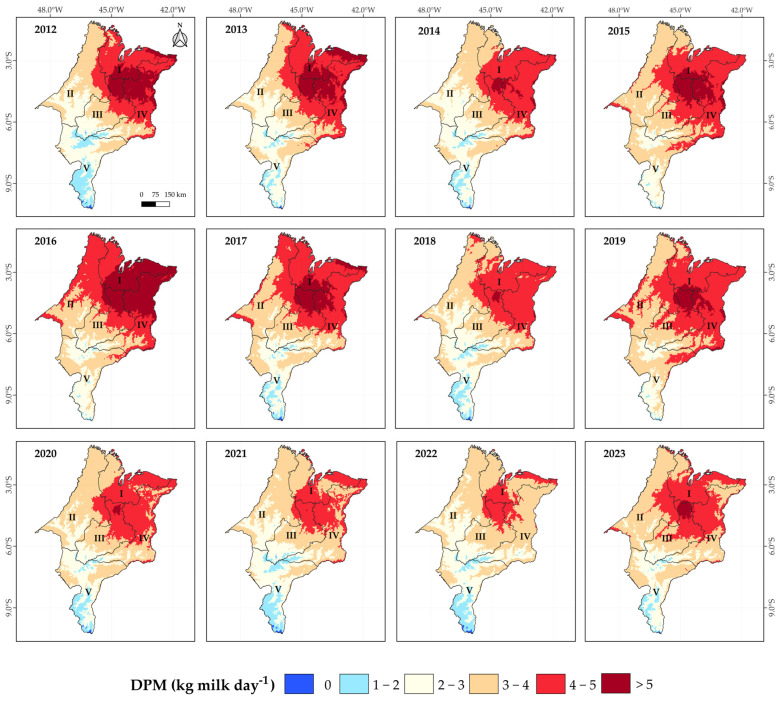
Kriging maps of the decrease in milk production (DMP) relative to the potential production capacity under thermal comfort conditions per cow: 25 kg day^−1^ cow^−1^ (PL25), from 2012 to 2023 in the mesoregions of Maranhão: I—North, II—West, III—Central, IV—East, and V—South.

Kriging maps illustrating the impact of heat stress on the decrease in milk production (DMP) in cows with a productive potential of 10 kg/day/cow (PL10) and 25 kg/day/cow (PL25) across the mesoregions of Maranhão, from 2012 to 2023, are presented in Figure 3 and Figure 4, respectively. For cows with PL10, the greatest impacts due to heat stress predominantly occurred in the Eastern and Northern mesoregions, with losses frequently exceeding 1.5 kg/cow/day. The years 2012, 2015, and especially 2016 stand out in this regard. On the other hand, the Southern mesoregion of Maranhão recorded lower productive losses, with reductions below 0.5 kg/cow/day (Figure 5). As expected, the impact of heat stress was even more pronounced in cows with higher productive potential, i.e., PL25 (Figure 6). A decreasing gradient of decrease in milk production (DPM) was observed from the northeast to the southwest of Maranhão. The Northern, Eastern, and parts of the Central mesoregions exhibited losses frequently exceeding 5 kg/cow/day, especially during years of higher thermal load (2012, 2013, 2016, and 2017). Furthermore, a significant temporal variation in the impact of heat stress was observed, with 2012 and 2016 standing out clearly. Although subsequent periods showed a relative reduction in the intensity of this impact, the Northern and East regions remained consistently vulnerable.

The impacts of heat stress on productivity were evident and, as expected, followed the pattern of THI values. Overall, we estimated losses frequently exceeding 1.5 kg/cow/day for PL10 cows and up to 5 kg/cow/day for PL25 cows in the thermally most challenging mesoregions of Maranhão identified earlier. In a study conducted with dairy cows in Bahia, Brazil [21], losses of up to 1 kg/cow/day for PL10 cows and up to 4.5 kg/cow/day for PL25 cows were estimated in the most critical areas (THI > 77). More recently, researchers carried out a bioclimatic zoning of the Central-West region and identified thermal comfort during autumn/winter (THI < 70), with no productivity losses [19]. However, in spring/summer, they reported losses of up to 2 kg/cow/day for PL25 cows in the southern part of the region, with no impacts observed for PL10 cows.

The bioclimatic forecasts presented in this study have practical applications for various public and private institutions involved in agricultural planning and climate adaptation in Maranhão. State-level agencies such as the Agência Estadual de Pesquisa Agropecuária e Extensão Rural do Maranhão (AGERP), the Agência Estadual de Defesa Agropecuária do Maranhão (AGED), and the State Secretariat of Agriculture can use these projections to guide mitigation strategies, training programs, and policy development. In addition, national institutions like the Serviço Nacional de Aprendizagem Rural (SENAR), the Ministry of Agriculture and Livestock (MAPA), and research units of the Brazilian Agricultural Research Corporation (EMBRAPA) may incorporate these findings into technical assistance models and innovation hubs focused on livestock climate resilience. The academic sector can support these initiatives by offering data analysis, educational outreach, and the co-creation of region-specific adaptation frameworks, thus ensuring the effective transfer of scientific knowledge to decision-making processes and rural communities.

Given the increasing prevalence of misinformation, particularly in animal science, disseminating scientific findings through social media platforms (e.g., Twitter (X), Facebook, and Instagram) can facilitate knowledge transfer and public engagement. By strategically using these platforms, the results of this study can reach producers, policymakers, and the general public, raising awareness about climate-related risks to dairy production and promoting evidence-based decision-making. A study by Lamanna et al. [63] highlighted the power of social media in communicating complex topics, such as the impact of climate change on dairy cattle, to a broad audience. Such initiatives complement outreach efforts involving influencers and provide examples of how digital platforms can enhance engagement and raise awareness within specialized fields like climate-smart livestock farming.

### 3.2. Future Scenarios (RCP 4.5 and 8.5)

**Figure 7:**
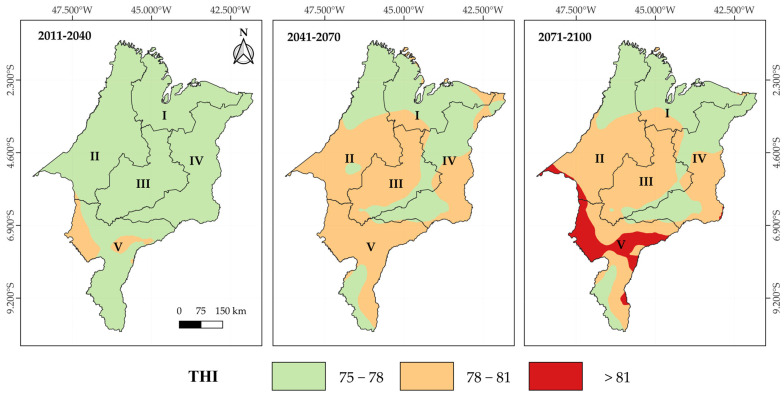
Kriging maps of future THI projections under the RCP4.5 scenarios for the short-term (2011–2040), medium-term (2041–2070), and long-term (2071–2100) periods in the mesoregions of Maranhão: I—North, II—West, III—Central, IV—East, and V—South.

Future climate projections make the scenario even more concerning. Figure 7 presents kriging maps of future THI projections under the RCP4.5 climate scenario for the short-term (2011–2040), mid-term (2041–2070), and long-term (2071–2100) periods across the mesoregions of Maranhão. It is observed that, in the short-term period (2011–2040), most of the state of Maranhão is expected to exhibit THI values ranging between 75 and 78. In the mid-term (2041–2070), an increase in heat stress levels is projected, with THI values predominantly ranging from 78 to 81 in the Southern, Central, and Western mesoregions, especially in the southwestern portion of the latter. These THI values correspond to the threshold of high heat stress for cattle (Table 1). In the long-term period (2071–2100), this pattern intensifies, with the formation of extensive areas presenting THI values above 81, mainly in the Southern and extreme southwestern regions of the state.

**Figure 8:**
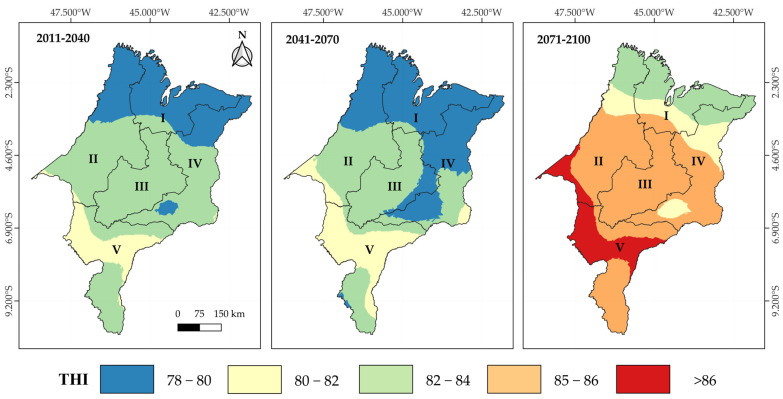
Kriging maps of future THI projections under the RCP8.5 scenarios for the short-term (2011–2040), medium-term (2041–2070), and long-term (2071–2100) periods in the mesoregions of Maranhão: I—North, II—West, III—Central, IV—East, and V—South.

Figure 8 presents kriging maps of future THI projections under the RCP8.5 climate scenario for the short-term (2011–2040), mid-term (2041–2070), and long-term (2071–2100) periods across the mesoregions of Maranhão. In this more pessimistic scenario, THI values between 82 and 84 are predominant across most of Maranhão in the short term (2011–2040), although a small area in the far north shows values between 78 and 80. In the mid term (2041–2070), this region expands, accompanied by intermediate zones with values ranging from 80 to 82. In the long term (2071–2100), a marked inversion in the thermal THI gradient is observed compared to the current pattern, shifting from an increasing north-to-south thermal load (Figure 2) to a decreasing distribution in the same direction. A wide area of Maranhão is projected to exhibit THI values above 86, a condition aligned with the threshold of thermal emergency (THI ≥ 84) for cattle according to the Livestock Weather Safety Index [41]. Under this scenario, livestock production will be severely compromised, especially in the absence of effective heat management strategies and adaptation to new climatic conditions.

**Figure 9:**
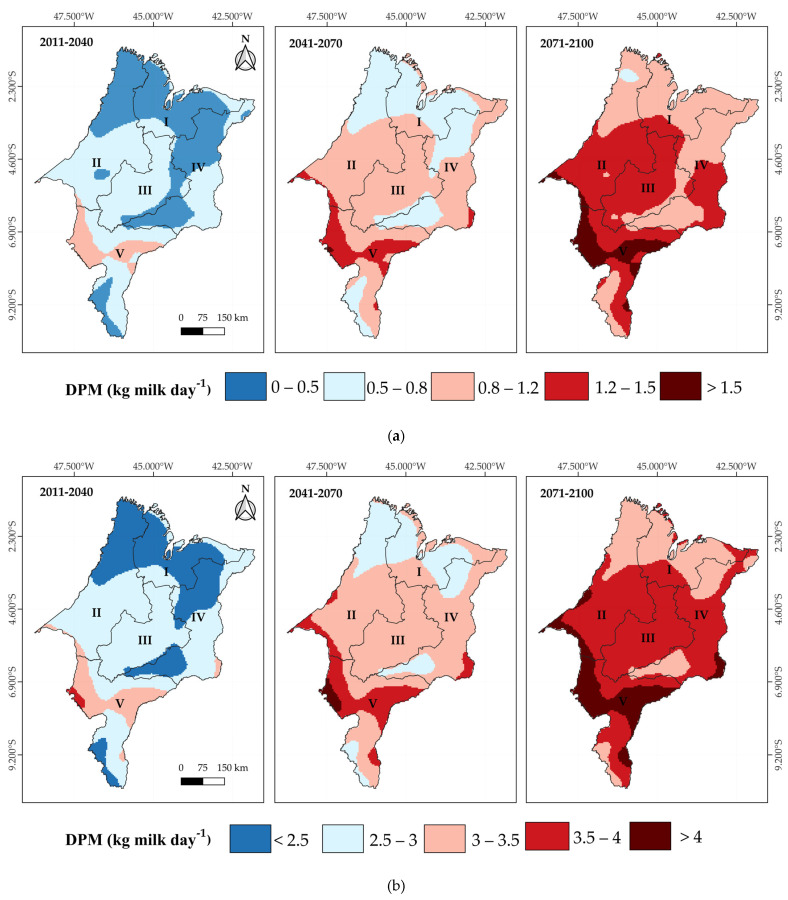
Kriging maps of the decrease in milk production (DMP) relative to the potential production capacity under thermal comfort conditions per cow: 10 kg day^−1^ cow^−1^ (PL10) (**a**) and 25 kg day^−1^ cow^−1^ (PL25) (**b**), based on future THI projections under the RCP4.5 scenario for the short-term (2011–2040), medium-term (2041–2070), and long-term (2071–2100) periods in the mesoregions of Maranhão: I—North, II—West, III—Central, IV—East, and V—South.

**Figure 10:**
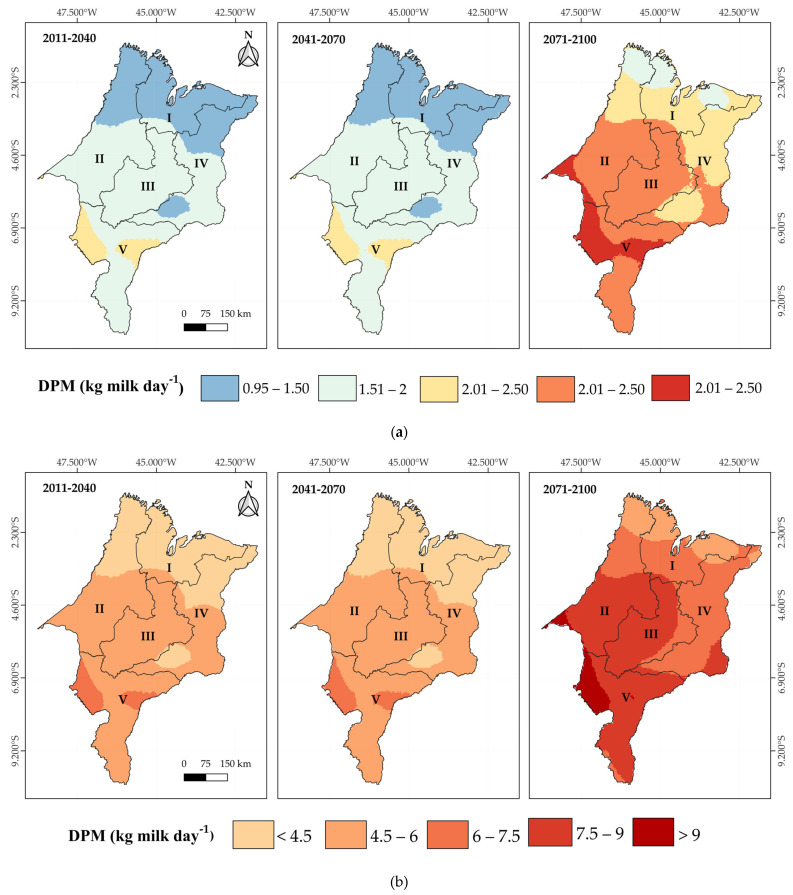
Kriging maps of the decrease in milk production (DMP) relative to the potential production capacity under thermal comfort conditions per cow: 10 kg day^−1^ cow^−1^ (PL10) (**a**) and 25 kg day^−1^ cow^−1^ (PL25) (**b**), based on future THI projections under the RCP8.5 scenario for the short-term (2011–2040), medium-term (2041–2070), and long-term (2071–2100) periods in the mesoregions of Maranhão: I—North, II—West, III—Central, IV—East, and V—South.

Under the RCP4.5 scenario, cows with PL10 are projected to lose, on average, 0.5 kg of milk per day relative to their productive potential in the short term. This impact will intensify over time, reaching losses of up to 1.2 kg of milk/cow/day across most of the state in the mid term (2041–2070) and exceeding 1.5 kg/cow/day in the long term (2071–2100), with the Central and Southern mesoregions being the most affected (Figure 9a). For PL25 cows, the losses are even more severe, surpassing 4 kg of milk per cow/day in the long term, with a strong impact on the Central, Western, and Southern mesoregions (Figure 9b), which are the main dairy production hubs in Maranhão. In the more pessimistic RCP8.5 scenario, projected losses for PL10 cows’ range between 1.5 and 2 kg of milk/day by the mid term (2041–2070). However, these losses become more significant in the long term (2071–2100), exceeding 2 kg/cow/day, especially in the Western, Central, and Southern mesoregions (Figure 10a). For PL25 cows, losses exceed 4.5 kg of milk/day as early as the short term and progressively intensify, reaching values above 9 kg/cow/day in the long term (Figure 10b).

It is worth noting that the projected increases in the THI could generate significant ecological and economic impacts in Maranhão. High environmental heat loads can reduce pasture quality, alter land use patterns, and promote biodiversity loss through vegetation shifts [3,60,64,65]. Economically, dairy productivity may decline due to reduced milk yields, as already documented, but also because of fertility issues and increased animal health costs [13,15]. Smallholder farmers are particularly vulnerable, given their limited access to mitigation technologies [31,34]. These dynamics may lead to shifts in livelihood strategies and rural exodus. Integrating climate-resilient practices and adaptive policies is essential to sustain dairy systems under future climate scenarios.

The results of this study corroborate previous research. Studies conducted in different parts of the world have reinforced the severity of climate change impacts on livestock production systems. In East Africa, it was estimated that under the RCP8.5 scenario, more than 50% of the days between 2071 and 2100 will be under severe heat stress conditions for dairy cattle [66]. In the United Kingdom, significant increases in the frequency of days with THI values above 70 have been projected, potentially exceeding 90 days per year by 2100, especially in southern and eastern England [25]. In Brazil, studies investigating the impacts of climate change on heat stress in livestock under the pessimistic SSP5-8.5 scenario identified a substantial increase in the number of days with high heat stress in the South and Southeast regions, and with extreme heat stress in the North and Central-West regions [22]. Projections for the 2061–2080 period indicate that these regions may face up to 200 days per year under extreme heat stress conditions.

However, the impact of climate change on dairy production is a multifactorial phenomenon involving complex interactions between physiological, environmental, genetic, and management factors [14,67,68]. Therefore, exclusive reliance on the THI for predictions can be limiting, as unifactorial analyses do not capture the full complexity of heat stress in dairy cattle [17,69]. Although widely used, the THI only considers ambient temperature and relative humidity, overlooking environmental variables such as solar radiation, wind, and accumulated heat load, as well as animal behavioral aspects like shade-seeking [11,17]. Furthermore, the use of regionalized climate datasets may smooth out local extremes and specific microclimates, leading to underestimation or overestimations of risk in certain areas. Thus, future studies should integrate multidimensional modeling frameworks, combining the THI with indices that include solar radiation (e.g., Heat Load Index—HLI, and Comprehensive Climate Index—CCI), geno-type-environment interactions, management practices, and socioeconomic factors.

On the other hand, the present study provides genuine and relevant information for the formulation of mitigation and adaptation strategies in the face of the challenges imposed on dairy production by climate change. The results of this study highlight the urgent need for the implementation of scientifically based mitigation and adaptation strategies, such as smart environmental management, genetic improvement of animals with greater heat tolerance, and the strengthening of public policies aimed at supporting small-scale farmers. In this context, the importance of continuous and regionally tailored monitoring of bioclimatic indicators stands out, enabling the anticipation of critical scenarios and more effective decision-making in the productive sector. With projections indicating an increase in drought and heat conditions across much of Brazilian territory [22,56], heat stress on livestock is expected to intensify, posing an additional challenge to food security in the context of global climate change. These findings are aligned with the Plano Maranhão 2050, a strategic long-term initiative that emphasizes sustainable development, climate resilience, and rural inclusion through innovation and effective governance. The incorporation of bioclimatic forecasting into the plan’s implementation could contribute to more climate-resilient agricultural systems in the region.

## 4. Conclusions

We identified a decreasing north–south thermal gradient, associated with the magnitude of temporal variability influenced by the Intertropical Convergence Zone and the effects of the El Niño–Southern Oscillation phenomena. Current milk production losses were frequently estimated to exceed 1.5 kg/cow/day and 5 kg/cow/day for cows with PL10 and PL25, respectively.

Climate projections indicate a progressive worsening of thermal conditions under the RCP4.5 and RCP8.5 scenarios, making currently productive areas potentially unviable for dairy farming and directly threatening regional food security. Estimated milk production losses may reach 4 and 9 kg/cow/day for PL25 cows under the RCP4.5 and RCP8.5 scenarios, respectively, significantly compromising the sustainability of livestock systems. Furthermore, traditionally more resilient areas, such as the Southern mesoregion, are projected to become critical heat stress zones.

The Standardized Precipitation Index proved to be a promising tool for identifying spatial patterns of THI, and its correlation with heat stress represents a novel methodological advancement in the literature. This can contribute to a better understanding of the interaction between bioclimatic variability and animal thermal discomfort.

## Figures and Tables

**Table 1:** THI onset of the stress level for dairy cattle, beef cattle, and general cattle, classified as moderate, high, and extreme heat stress.

Item	Onset of the Stress Level			References
	Moderate	High	Extreme	
General cattle	72	78	90	Fuquay [42]
Cattle dairy	72	79	89	Pinto et al. [16] and Rahimi et al. [3]

**Table 2:** Classification of the Standardized Precipitation Index (SPI).

SPI	Classification
≥2.00	Extremely Wet
1.00 to 1.99	Very Wet
0.50 to 0.99	Moderately Wet
0.49 to −0.49	Near Normal
−0.50 to −0.99	Moderately Dry
−1.00 to −1.99	Very Dry
≤−2.00	Extremely Dry

McKee et al. [47].

**Table 3:** Summary of descriptive statistics for annual THI values.

Year	Mean	Min ^1^	Max ^2^	SD ^3^	CV ^4^
2012	77.811	72.457	81.375	1.791	2.302
2013	78.030	72.934	81.346	1.642	2.104
2014	77.599	72.692	81.029	1.475	1.901
2015	78.263	73.393	81.425	1.307	1.670
2016	78.584	73.778	81.817	1.579	2.009
2017	78.178	72.885	81.549	1.536	1.965
2018	77.612	72.638	80.753	1.345	1.733
2019	78.373	73.690	81.229	1.172	1.495
2020	77.591	72.579	80.435	1.240	1.598
2021	77.265	72.179	89.099	1.273	1.648
2022	77.117	71.980	79.748	1.131	1.467
2023	77.791	73.088	80.809	1.204	1.548

^1^ Minimum. ^2^ Maximum. ^3^ Standard deviation. ^4^ Coefficient of variation.

**Table 4:** Cross-validation for the tested geostatistical models for annual THI values.

Spherical					
Year	ME ^1^	RMSE ^2^	MSE ^3^	RMSSE ^4^	ASE ^5^
2012	−0.000320986	0.128319447	−0.000512021	0.800392631	0.160438056
2013	−0.000315744	0.128538646	−0.000487145	0.81826913	0.157218868
2014	−0.000321951	0.128419579	−0.000554609	0.830047674	0.154838422
2015	−0.000338638	0.128918717	−0.000686003	0.846295409	0.152458347
2016	−0.000309894	0.128365609	−0.000448229	0.821033577	0.156453329
2017	−0.000304233	0.128152743	−0.000454579	0.828461322	0.154829967
2018	−0.000202561	0.130590697	0.000138732	0.791902598	0.164944244
2019	−0.000162532	0.133705573	0.000323739	0.767968754	0.17411815
2020	−0.000130996	0.133960782	0.000503925	0.771984532	0.173539787
2021	−0.000141931	0.133116484	0.000487361	0.76332796	0.174411679
2022	−1.051 × 10^−4^	0.133694996	0.000651064	0.748842232	0.178522544
2023	−0.000238688	0.130228631	−3.58 × 10^−5^	0.805170026	0.161808438
Gaussian					
Year	ME	RMSE	MSE	RMSSE	ASE
2012	0.001626452	0.182013053	0.00779711	0.802128	0.226911639
2013	0.001406824	0.185973639	0.00646772	0.778031648	0.23899667
2014	0.001205019	0.187393487	0.005395297	0.754600831	0.248276242
2015	0.001114403	0.190652883	0.004834541	0.7420926	0.2568169
2016	0.001339842	0.186079898	0.006101088	0.765508237	0.24303419
2017	0.00111343	0.189309448	0.004984191	0.75911917	0.2493122
2018	0.000599605	0.191581318	0.002662496	0.70881721	0.270140782
2019	0.000869422	0.181607237	0.003934458	0.705810768	0.257203767
2020	0.000660389	0.192203203	0.002877935	0.7045586	0.27267361
2021	0.001002446	0.189840465	0.00433775	0.721433355	0.263044782
2022	0.000106874	0.193464814	0.000758881	0.657260615	0.294165691
2023	0.001654166	0.179921759	0.007887942	0.78423291	0.229410673
Exponential					
Year	ME	RMSE	MSE	RMSSE	ASE
2012	−0.000472255	0.12889224	−0.000958666	0.582564257	0.221274032
2013	−0.000490464	0.129299708	−0.001043384	0.594798666	0.217402355
2014	−0.000490748	0.128962683	−0.001096212	0.610484416	0.21128051
2015	−0.000499266	0.129465871	−0.001163473	0.616646626	0.209993647
2016	−0.000458259	0.128978477	−0.000921173	0.602824793	0.213965415
2017	−0.000467414	0.128788414	−0.000981837	0.602542766	0.213797067
2018	−0.000458935	0.128393832	−0.00097723	0.624536891	0.20560194
2019	−0.000527456	0.12990522	−0.001237933	0.605038327	0.214717179
2020	−0.000469978	0.129369522	−0.00104148	0.641470262	0.201734207
2021	−0.000506541	0.129350044	−0.001099826	0.608753309	0.212502554
2022	−0.00047885	0.128458003	−0.001030727	0.625094344	0.205531904
2023	−0.000471804	0.129069315	−0.000974172	0.608991685	0.211972383

^1^ Mean error. ^2^ Root mean square error. ^3^ Mean square error. ^4^ Root mean square standardized error. ^5^ Average standardized error.

**Table 5:** Parameters for the fitted semivariogram models and degree of spatial dependence.

Year	Model	Nugget Effect	Sill	Range	^1^ DSD
2012	Spherical	0.00001	0.3991	7000	0.002
2013	Spherical	0.00001	0.3284	6000	0.003
2014	Spherical	0.000196	0.2993	5700	0.065
2015	Spherical	0.00001	0.4120	8000	0.002
2016	Spherical	0.00001	0.2980	5500	0.003
2017	Spherical	0.00001	0.3503	6600	0.002
2018	Spherical	0.004348	0.3363	7000	1.292
2019	Spherical	0.007121	0.4263	9000	1.670
2020	Spherical	0.007842	0.3599	8000	2.178
2021	Spherical	0.007233	0.3546	7500	2.039
2022	Spherical	0.009122	0.3087	6800	2.954
2023	Spherical	0.002712	0.4027	8000	0.673

^1^ Degree of spatial dependence (%).

## Data Availability

Publicly available datasets were analyzed in this study. These data can be found here: https://climateengine.com (accessed on 10 February 2025) and http://etamodel.cptec.inpe.br/ (accessed on 20 February 2025).

## Supplementary Materials

The following supporting information can be downloaded at: https://www.mdpi.com/article/10.3390/ani15111646/s1. Table S1: Summary of descriptive statistics for annual mean air temperature values; Table S2: Summary of descriptive statistics for annual wind speed values; Table S3: Cross-validation for the tested geostatistical models for annual mean air temperature values; Table S4: Cross-validation for the tested geostatistical models for annual wind speed values; Figure S1: Experimental semivariograms and cross-validation of kriging maps of THI from 2012 to 2023 in the mesoregions of Maranhão; Figure S2: Kriging maps of air temperature values from 2012 to 2023 in the mesoregions of Maranhão: I—North, II—West, III—Central, IV—East, and V—South; Figure S3: Kriging maps of wind speed values from 2012 to 2023 in the mesoregions of Maranhão: I—North, II—West, III—Central, IV—East, and V—South; Figure S4: Experimental semivariograms and cross-validation of kriging maps of air temperature from 2012 to 2023 in the mesoregions of Maranhão; Figure S5: Experimental semivariograms and cross-validation of kriging maps of wind speed from 2012 to 2023 in the mesoregions of Maranhão; Figure S6: Kriging maps of future air temperature projections under the RCP4.5 (a) and RCP8.5 (b) scenarios for the short-term (2011–2040), medium-term (2041–2070), and long-term (2071–2100) periods in the mesoregions of Maranhão: I—North, II—West, III—Central, IV—East, and V—South; Figure S7: Kriging maps of future wind speed projections under the RCP4.5 (a) and RCP8.5 (b) scenarios for the short-term (2011–2040), medium-term (2041–2070), and long-term (2071–2100) periods in the mesoregions of Maranhão: I—North, II—West, III—Central, IV—East, and V—South; Figure S8: Kriging maps of Standardized Precipitation Index (SPI) from 2012 to 2023 in the mesoregions of Maranhão: I—North, II—West, III—Central, IV—East, and V—South.
