# Supplementary material for: Bioclimatic Zoning and Climate Change Impacts on Dairy Cattle in Maranhão, Brazil
Source: Animals (Basel). 2025 Jun 3;15(11):1646. doi: 10.3390/ani15111646 (PMC12153783; doi:10.3390/ani15111646)

**Supplementary Materials**

# Bioclimatic Zoning and Climate Change Impacts on Dairy Cattle in Maranhão, Brazil

Andressa Carvalho de Sousa [1], Andreza Maciel de Sousa [1], Wellington Cruz Corrêa [1], Jordânio Inácio Marques [1], Kamila Cunha de Meneses [1], Héliton Pandorfi [2], Thieres George Freire da Silva [2], Jhon Lennon Bezerra da Silva [3], Marcos Vinícius da Silva [1] and Nítalo André Farias Machado [1*]

[1]Chapadinha Science Center, Federal University of Maranhão, Chapadinha 65500-000, Brazil;

[2]Department of Agricultural Engineering, Federal Rural University of Pernambuco, Recife 52171-900, Brazil

[3]Cerrado Irrigation Graduate Program, Goiano Federal Institute, Ceres 76300-000, Goiás, Brazil.

*Correspondence: nitalo.farias@ufma.br

Table S1

Summary of descriptive statistics for annual mean air temperature values.

| Year | Mean | Min[1] | Max[2] | SD[3] | CV[4] |
|---|---|---|---|---|---|
| 2012 | 26.889 | 23.450 | 29.137 | 1.132 | 4.208 |
| 2013 | 27.033 | 23.791 | 29.120 | 1.0372 | 3.837 |
| 2014 | 26.755 | 23.591 | 28.920 | 0.941 | 3.517 |
| 2015 | 27.163 | 24.049 | 29.154 | 0.826 | 3.041 |
| 2016 | 27.374 | 24.291 | 29.416 | 0.996 | 3.638 |
| 2017 | 27.110 | 23.508 | 29.241 | 0.977 | 3.604 |
| 2018 | 26.786 | 23.554 | 28.783 | 0.869 | 3.244 |
| 2019 | 27.281 | 24.308 | 29.070 | 0.734 | 2.691 |
| 2020 | 26.780 | 23.537 | 28.583 | 0.798 | 2.980 |
| 2021 | 26.583 | 23.295 | 28.333 | 0.814 | 3.062 |
| 2022 | 26.468 | 23.133 | 28.141 | 0.729 | 2.754 |
| 2023 | 26.886 | 23.875 | 28.783 | 0.761 | 2.830 |

[1]Minimum. [2]Maximum. [3]Standard deviation. [4]Coefficient of variation.

Table S2

Summary of descriptive statistics for annual wind speed values.

| Year | Mean | Min[1] | Max[2] | SD[3] | CV[4] |
|---|---|---|---|---|---|
| 2012 | 1.467 | 1.092 | 2.729 | 0.265 | 18.064 |
| 2013 | 1.425 | 1.079 | 2.493 | 0.250 | 17.544 |
| 2014 | 1.484 | 1.201 | 2.475 | 0.228 | 15.364 |
| 2015 | 1.596 | 1.126 | 2.675 | 0.286 | 17.920 |
| 2016 | 1.503 | 1.146 | 2.47 | 0.249 | 16.567 |
| 2017 | 1.574 | 1.290 | 2.352 | 0.184 | 11.690 |
| 2018 | 1.294 | 0.841 | 2.500 | 0.284 | 21.947 |
| 2019 | 1.169 | 0.825 | 2.241 | 0.216 | 18.477 |
| 2020 | 1.231 | 0.758 | 1.833 | 0.193 | 15.678 |
| 2021 | 1.147 | 0.841 | 1.908 | 0.180 | 15.693 |
| 2022 | 1.334 | 0.908 | 1.825 | 0.185 | 13.868 |
| 2023 | 1.414 | 1.100 | 2.058 | 0.177 | 12.518 |

[1]Minimum. [2]Maximum. [3]Standard deviation. [4]Coefficient of variation.

Table S3

Cross-validation for the tested geostatistical models for annual mean air temperature values

| Spherical | | | | | |
|---|---|---|---|---|---|
| Year | ME[1] | RMSE[2] | MSE[3] | RMSSE[4] | ASE[5] |
| 2012 | -0.000205305 | 0.082211661 | -0.000533553 | 0.801209757 | 0.102679483 |
| 2013 | -0.000200358 | 0.082172423 | -0.00050428 | 0.826236232 | 0.099534103 |
| 2014 | -0.000204936 | 0.082198787 | -0.000587313 | 0.855976353 | 0.096105142 |
| 2015 | -0.000215991 | 0.082135197 | -0.000712451 | 0.848934244 | 0.096825771 |
| 2016 | -0.0001938 | 0.081797651 | -0.000454343 | 0.841799328 | 0.09723369 |
| 2017 | -0.000193494 | 0.081890327 | -0.000473753 | 0.840975162 | 0.097459257 |
| 2018 | -0.000120973 | 0.084084031 | 0.000217326 | 0.791784334 | 0.10621371 |
| 2019 | -0.000109248 | 0.084923205 | 0.000252636 | 0.773825547 | 0.109753444 |
| 2020 | $-8.19 \times 10^{-5}$ | 0.085825575 | 0.00049992 | 0.771998349 | 0.111175679 |
| 2021 | $-9.28 \times 10^{-5}$ | 0.085384528 | 0.000452708 | 0.766104395 | 0.111463969 |
| 2022 | $-2.79 \times 10^{-5}$ | 0.087383584 | 0.000913714 | 0.736658549 | 0.118584279 |
| 2023 | -0.00013748 | 0.083618374 | $7.63 \times 10^{-5}$ | 0.803959345 | 0.104037462 |
| Gaussian | | | | | |
| Year | ME | RMSE | MSE | RMSSE | ASE |
| 2012 | 0.000835655 | 0.121424173 | 0.005867511 | 0.776606145 | 0.156301448 |
| 2013 | 0.00045521 | 0.123366644 | 0.003089659 | 0.72152616 | 0.170883013 |
| 2014 | 0.000441129 | 0.123315133 | 0.002978388 | 0.714932486 | 0.17238961 |
| 2015 | 0.000473844 | 0.123462419 | 0.003144899 | 0.712593309 | 0.173154822 |
| 2016 | 0.000557042 | 0.122305438 | 0.003815208 | 0.733422053 | 0.166676007 |
| 2017 | 0.000451164 | 0.122702137 | 0.003099707 | 0.723840617 | 0.169433035 |
| 2018 | 0.000308054 | 0.123952861 | 0.002098401 | 0.674811211 | 0.183565326 |
| 2019 | 0.000290605 | 0.123865886 | 0.001968925 | 0.674745404 | 0.183452757 |
| 2020 | 0.000195298 | 0.124201639 | 0.00145892 | 0.681010733 | 0.182270527 |
| 2021 | 0.000418079 | 0.123312678 | 0.002823683 | 0.69778248 | 0.176626158 |
| 2022 | $9.47 \times 10^{-5}$ | 0.124168571 | 0.000899863 | 0.663017223 | 0.187158578 |
| 2023 | 0.000229171 | 0.124237606 | 0.001628482 | 0.664802599 | 0.186752305 |
| Exponential | | | | | |
| Year | ME | RMSE | MSE | RMSSE | ASE |
| 2012 | -0.000288656 | 0.082496403 | -0.00089806 | 0.586042298 | 0.140792737 |

| | | | | | |
|---|---|---|---|---|---|
| 2013 | -0.000270525 | 0.082409439 | -0.000803901 | 0.602293302 | 0.136877256 |
| 2014 | -0.00030466 | 0.082549167 | -0.001057929 | 0.613082836 | 0.134672248 |
| 2015 | -0.000297301 | 0.082380439 | -0.001053532 | 0.621410071 | 0.132610451 |
| 2016 | -0.000280168 | 0.082103456 | -0.000880885 | 0.612841799 | 0.133985616 |
| 2017 | -0.000262758 | 0.082112712 | -0.00078651 | 0.615039712 | 0.133568466 |
| 2018 | -0.000282375 | 0.082416766 | -0.000895012 | 0.626294565 | 0.131624318 |
| 2019 | -0.000292956 | 0.082448963 | -0.001021571 | 0.626530991 | 0.131634996 |
| 2020 | -0.00027494 | 0.082684695 | -0.000902094 | 0.653010965 | 0.126672239 |
| 2021 | -0.000269293 | 0.082713603 | -0.000814937 | 0.645746563 | 0.128146679 |
| 2022 | $-2.73 \times 10^{-4}$ | 0.082307145 | -0.000853912 | 0.649012204 | 0.126859435 |
| 2023 | -0.000267204 | 0.082358826 | -0.00080602 | 0.632910771 | 0.130168669 |

[1]Mean error. [2]Root mean squar error. [3]Mean square error. [4]Root mean square standardized error. [5]Average standardized error.

Table S4

Cross-validation for the tested geostatistical models for annual wind speed values

| | | Spherical | | | |
|---|---|---|---|---|---|
| Year | ME[1] | RMSE[2] | MSE[3] | RMSSE[4] | ASE[5] |
| 2012 | $2.51 \times 10^{-5}$ | 0.005888077 | 0.001016865 | 0.198555627 | 0.029397127 |
| 2013 | $2.46 \times 10^{-5}$ | 0.00587653 | 0.001072721 | 0.215033622 | 0.027094985 |
| 2014 | $2.22 \times 10^{-5}$ | 0.005855997 | 0.000967779 | 0.209550856 | 0.027700369 |
| 2015 | $2.73 \times 10^{-5}$ | 0.005884585 | 0.001091054 | 0.205882806 | 0.028339731 |
| 2016 | $2.39 \times 10^{-5}$ | 0.005860335 | 0.001018727 | 0.209409907 | 0.027749647 |
| 2017 | $1.61 \times 10^{-5}$ | 0.005861276 | 0.000789962 | 0.237867906 | 0.024436935 |
| 2018 | $2.43 \times 10^{-5}$ | 0.008635566 | 0.000798573 | 0.261035016 | 0.032946513 |
| 2019 | $6.39 \times 10^{-6}$ | 0.00848083 | 0.000556435 | 0.289241427 | 0.029177985 |
| 2020 | $3.23 \times 10^{-6}$ | 0.009472863 | 0.000368143 | 0.401070223 | 0.023564388 |
| 2021 | $9.11 \times 10^{-6}$ | 0.009118394 | 0.000605613 | 0.392121242 | 0.02316413 |
| 2022 | $8.33 \times 10^{-6}$ | 0.008826727 | 0.000520317 | 0.383088724 | 0.022949407 |
| 2023 | $7.41 \times 10^{-6}$ | 0.008842566 | $5.70 \times 10^{-4}$ | 0.38129127 | 0.023092005 |
| | | Gaussian | | | |
| Year | ME | RMSE | MSE | RMSSE | ASE |
| 2012 | $8.30 \times 10^{-5}$ | 0.007032114 | 0.010712931 | 0.903822264 | 0.007758878 |
| 2013 | $8.37 \times 10^{-5}$ | 0.007069659 | 0.010825993 | 0.912478366 | 0.007726391 |
| 2014 | $7.18 \times 10^{-5}$ | 0.006885916 | 0.009966881 | 0.958926431 | 0.007159693 |
| 2015 | $7.98 \times 10^{-5}$ | 0.007114556 | 0.010175624 | 0.906325581 | 0.007827267 |
| 2016 | $9.88 \times 10^{-5}$ | 0.007310013 | 0.011298487 | 0.829971511 | 0.008782574 |
| 2017 | 0.000102069 | 0.007936288 | 0.009186667 | 0.7034129 | 0.011251273 |
| 2018 | $9.58 \times 10^{-5}$ | 0.010063436 | 0.009442755 | 0.994902711 | 0.010100562 |
| 2019 | 0.000203545 | 0.01023285 | 0.015245186 | 0.752504658 | 0.013573351 |
| 2020 | 0.000129428 | 0.011655538 | 0.009488282 | 0.826237387 | 0.014091003 |
| 2021 | 0.000133187 | 0.011486578 | 0.009101525 | 0.762207342 | 0.015041559 |
| 2022 | $1.04 \times 10^{-4}$ | 0.010683676 | 0.007789744 | 0.78916468 | 0.013510756 |
| 2023 | 0.000117656 | 0.011040446 | 0.008136526 | 0.739386421 | 0.014901817 |
| | | Exponential | | | |
| Year | ME | RMSE | MSE | RMSSE | ASE |
| 2012 | $2.74 \times 10^{-5}$ | 0.005946378 | 0.000832785 | 0.143888602 | 0.040897069 |

| | | | | | |
|---|---|---|---|---|---|
| 2013 | $2.63 \times 10^{-5}$ | 0.005926303 | 0.000833988 | 0.15097519 | 0.038865499 |
| 2014 | $2.36 \times 10^{-5}$ | 0.005914032 | 0.000772875 | 0.15010959 | 0.038990755 |
| 2015 | $3.03 \times 10^{-5}$ | 0.005929303 | 0.000891817 | 0.14785225 | 0.039713333 |
| 2016 | $2.61 \times 10^{-5}$ | 0.005909295 | 0.000836927 | 0.152139202 | 0.038463578 |
| 2017 | $1.62 \times 10^{-5}$ | 0.005907867 | 0.000620755 | 0.17365505 | 0.033697348 |
| 2018 | $2.49 \times 10^{-5}$ | 0.008672124 | 0.000621573 | 0.18758295 | 0.045995513 |
| 2019 | $4.69 \times 10^{-7}$ | 0.008523516 | 0.000319591 | 0.209514754 | 0.040435716 |
| 2020 | $-3.94 \times 10^{-7}$ | 0.009490483 | 0.000195564 | 0.287952916 | 0.032870342 |
| 2021 | $6.90 \times 10^{-6}$ | 0.00913917 | 0.000407744 | 0.280767227 | 0.032411693 |
| 2022 | $6.25 \times 10^{-6}$ | 0.008852304 | 0.000353307 | 0.277726593 | 0.031729262 |
| 2023 | $5.67 \times 10^{-6}$ | 0.008868963 | 0.000400476 | 0.276020481 | 0.031976274 |

[1]Mean error. [2]Root mean squar error. [3]Mean square error. [4]Root mean square standardized error. [5]Average standardized error.

Figure S1

Experimental semivariograms and cross-validation of kriging maps of THI from 2012 to 2023 in the mesoregions of Maranhão.

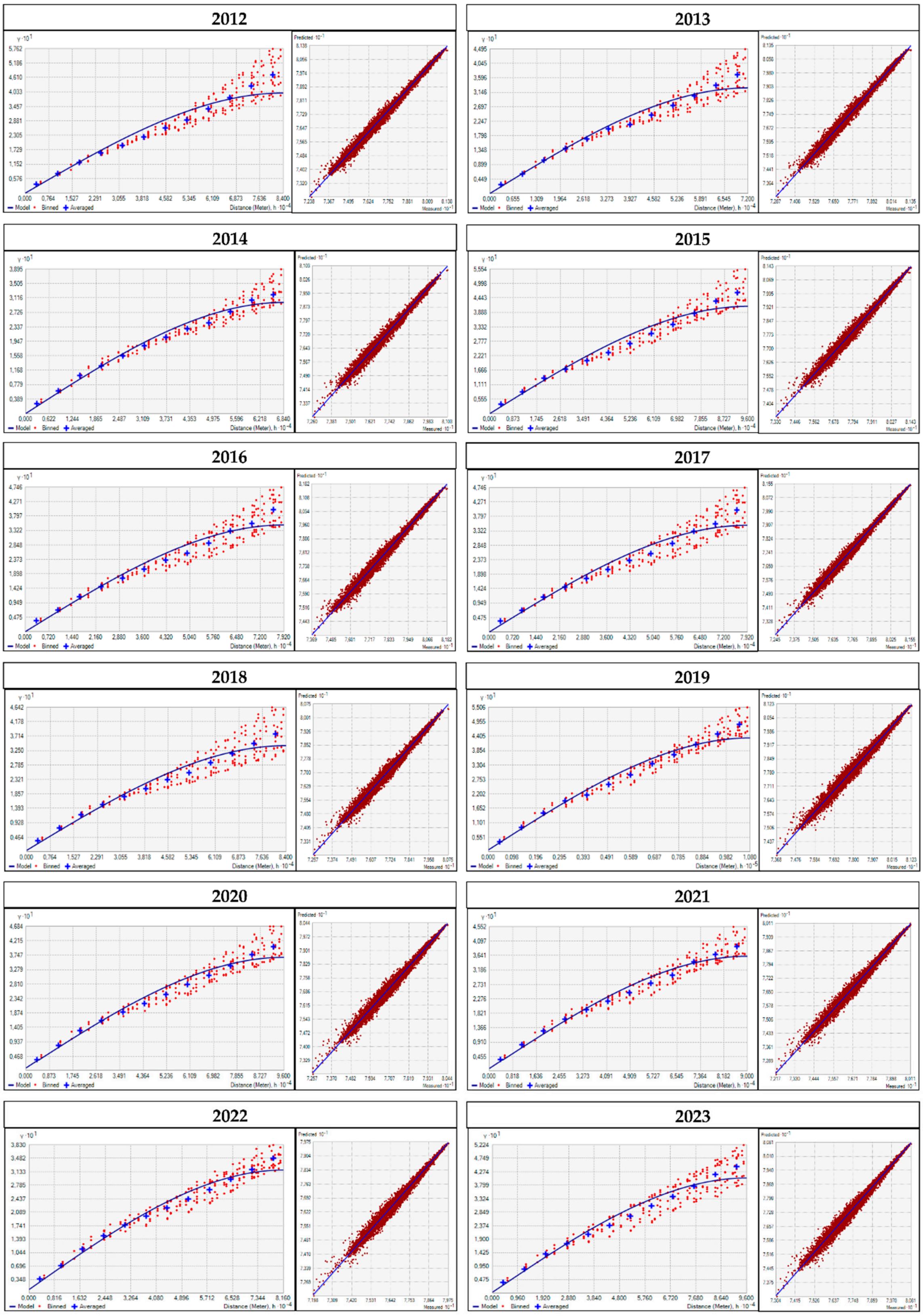

Figure S2

Kriging maps of air temperature values from 2012 to 2023 in the mesoregions of Maranhão: I—North, II—West, III—Central, IV—East, and V—South.

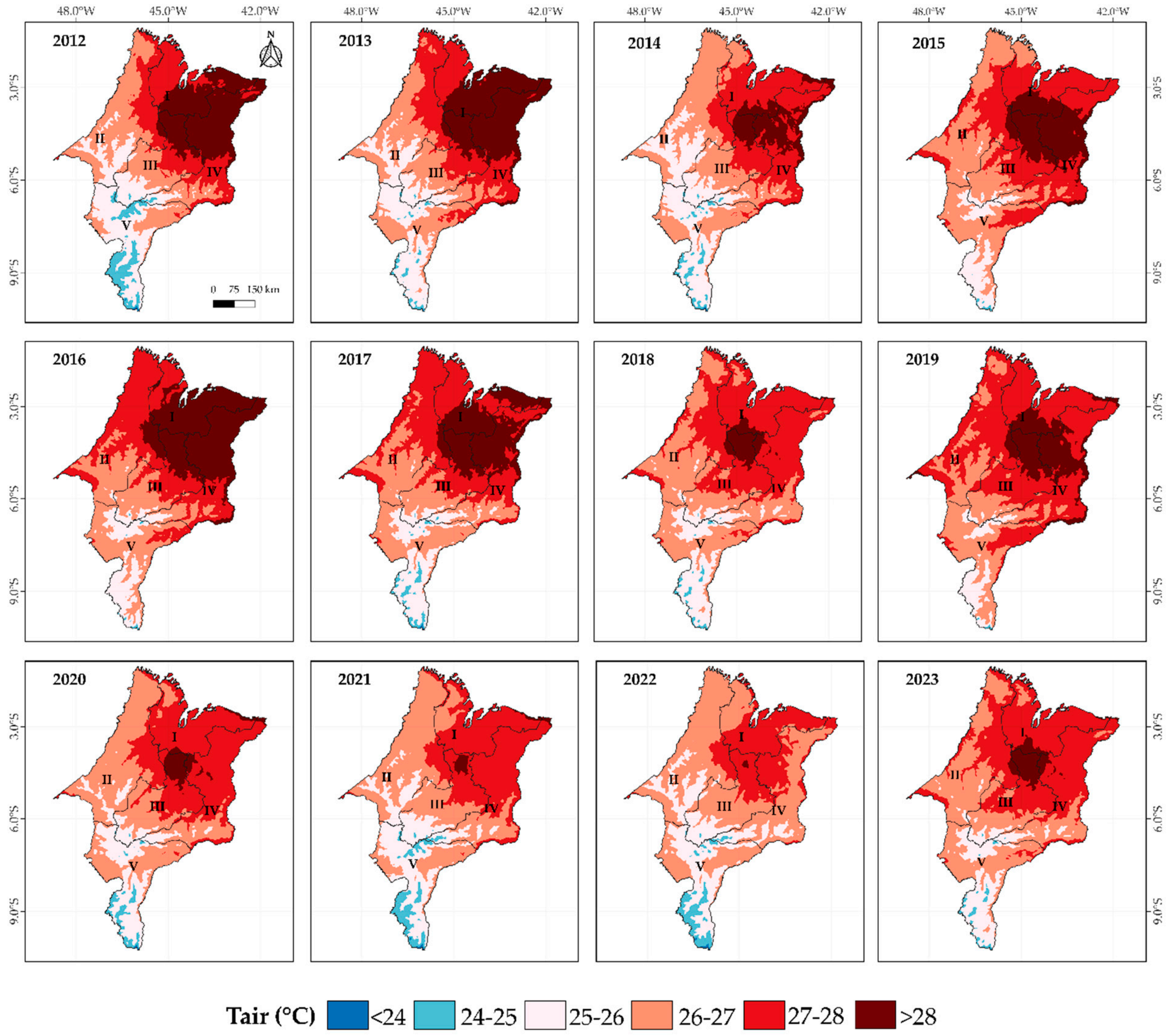

Figure S3

Kriging maps of wind speed values from 2012 to 2023 in the mesoregions of Maranhão: I—North, II—West, III—Central, IV—East, and V—South.

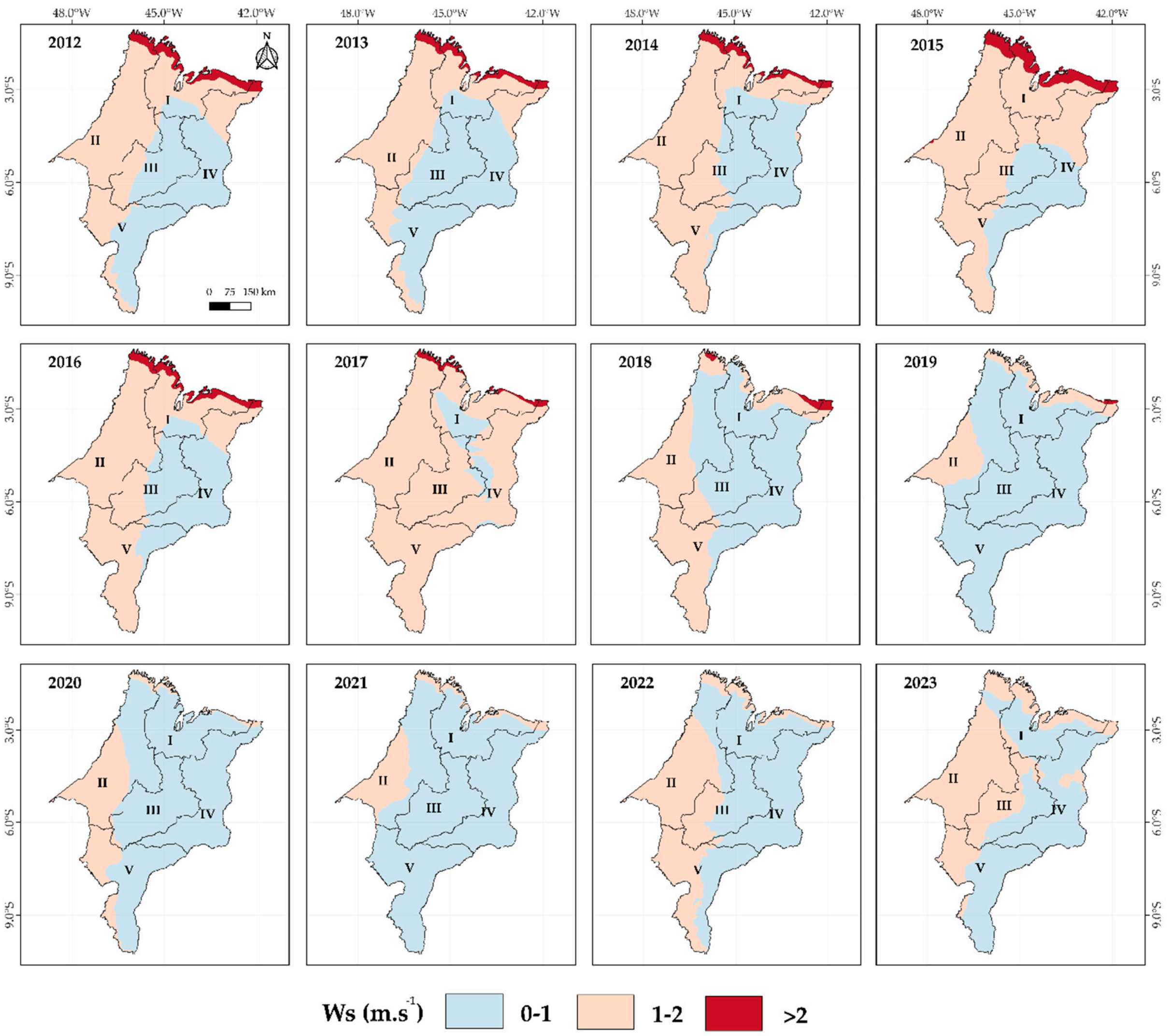

Figure S4

Experimental semivariograms and cross-validation of kriging maps of air temperature from 2012 to 2023 in the mesoregions of Maranhão.

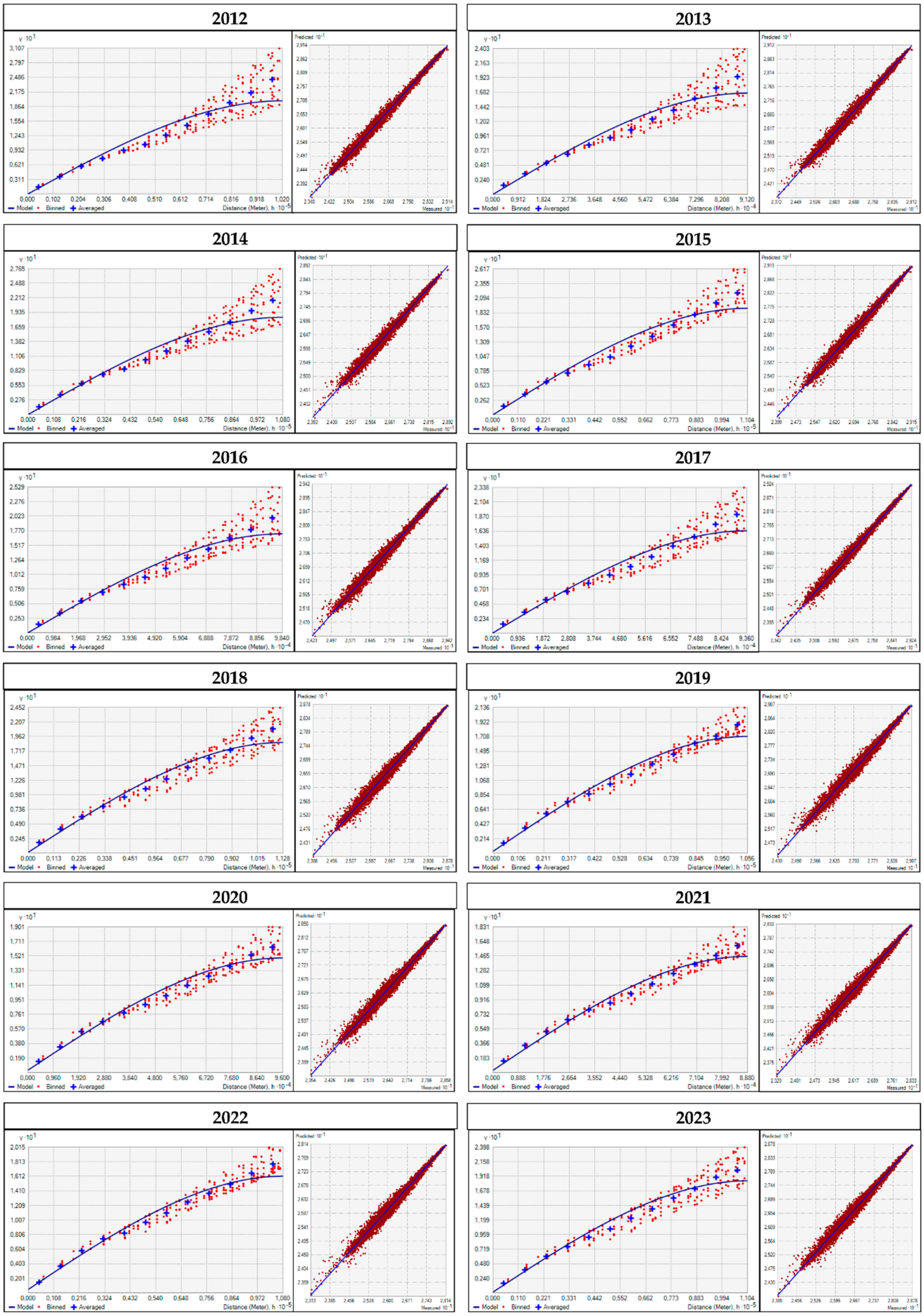

Figure S5

Experimental semivariograms and cross-validation of kriging maps of wind speed from 2012 to 2023 in the mesoregions of Maranhão.

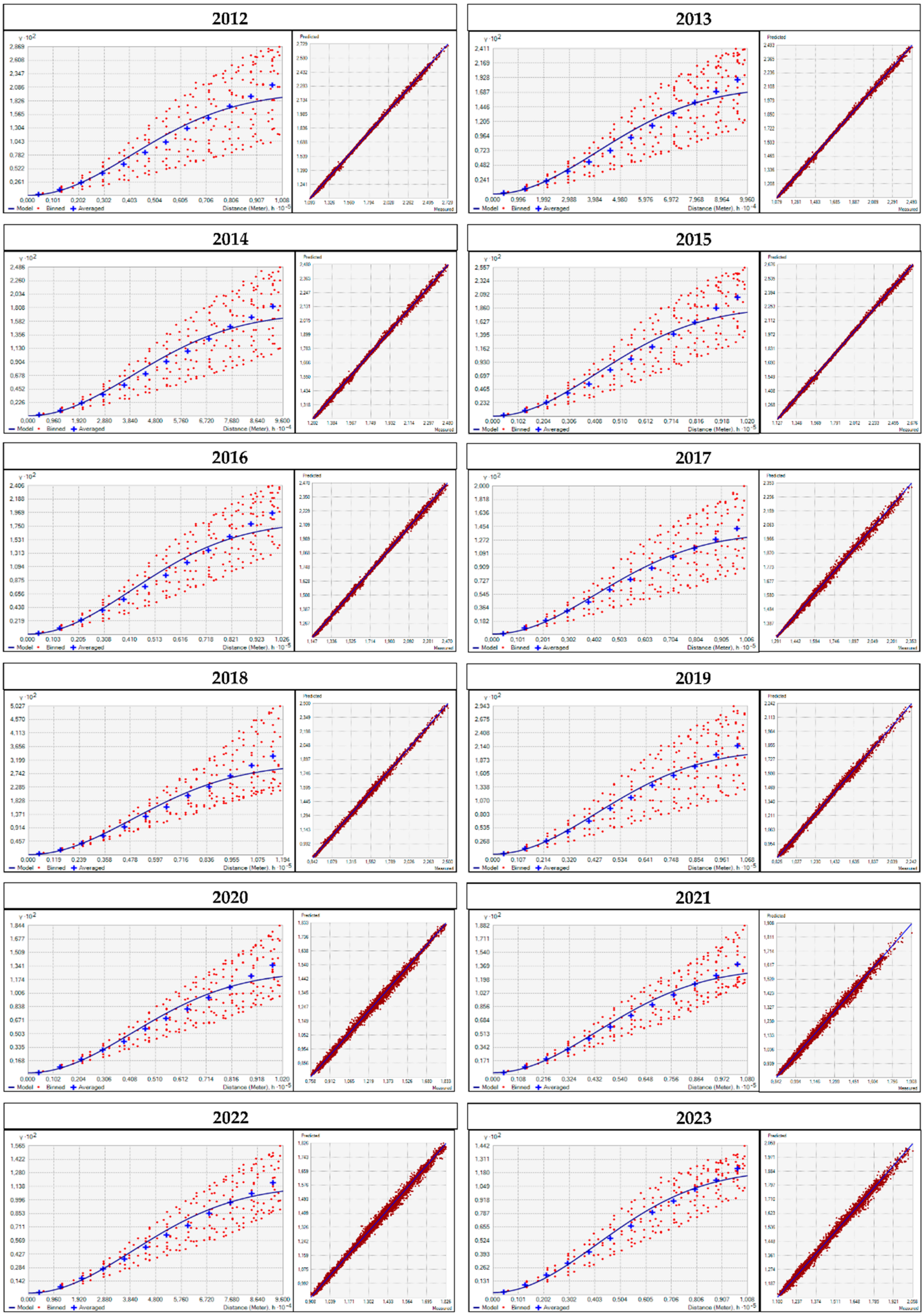

Figure S6

Kriging maps of future air temperature projections under the RCP4.5 (a) and RCP8.5 (b) scenarios for the short-term (2011–2040), medium-term (2041–2070), and long-term (2071–2100) periods in the mesoregions of Maranhão: I—North, II—West, III—Central, IV—East, and V—South.

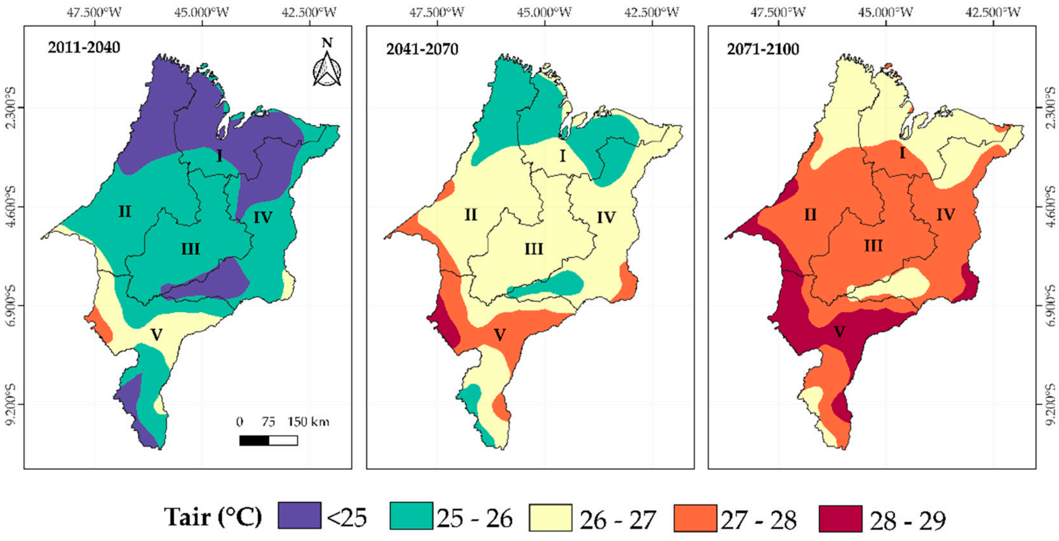


(a)

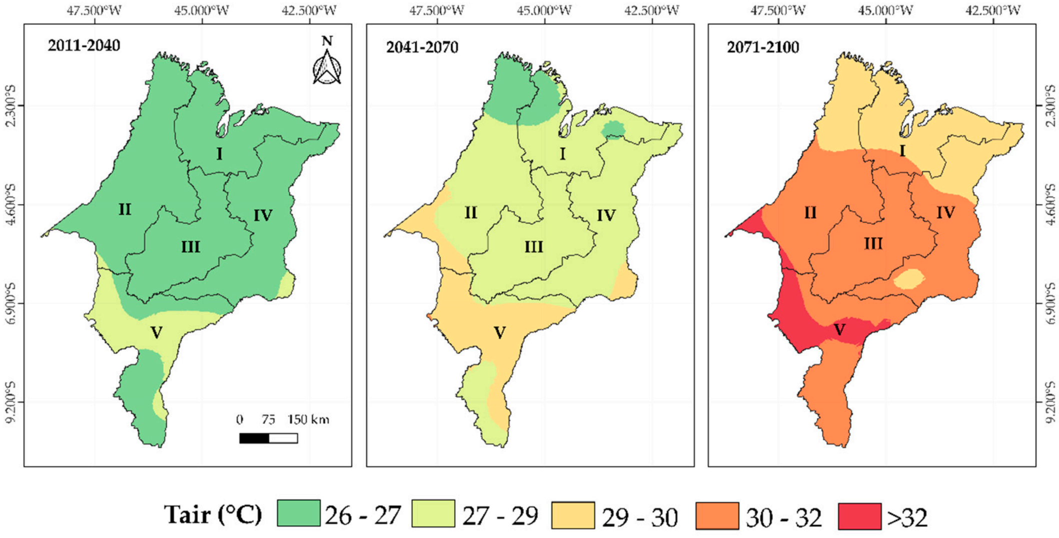


(b)

Figure S7

Kriging maps of future wind speed projections under the RCP4.5 (a) and RCP8.5 (b) scenarios for the short-term (2011–2040), medium-term (2041–2070), and long-term (2071–2100) periods in the mesoregions of Maranhão: I—North, II—West, III—Central, IV—East, and V—South.

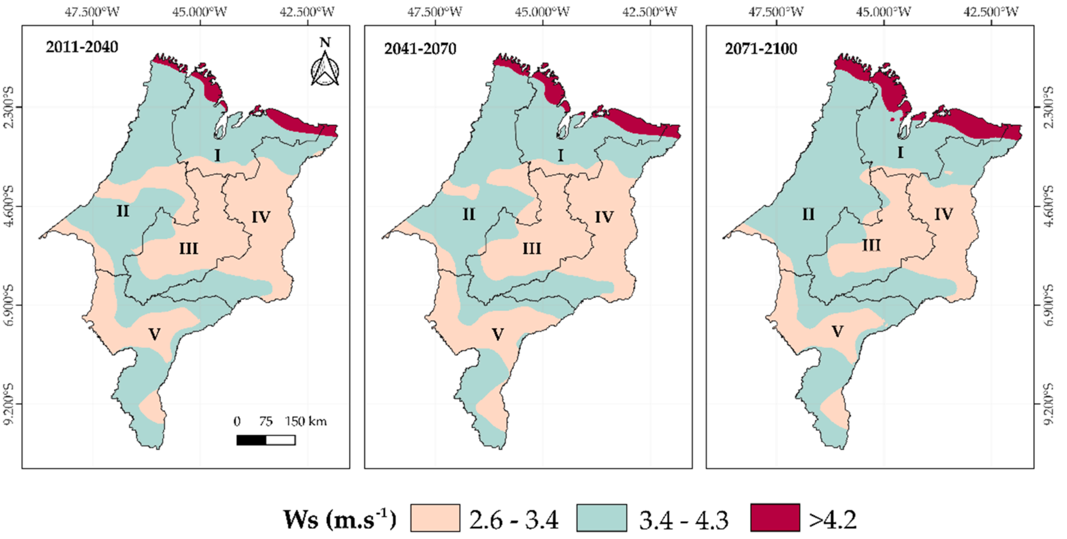


(a)

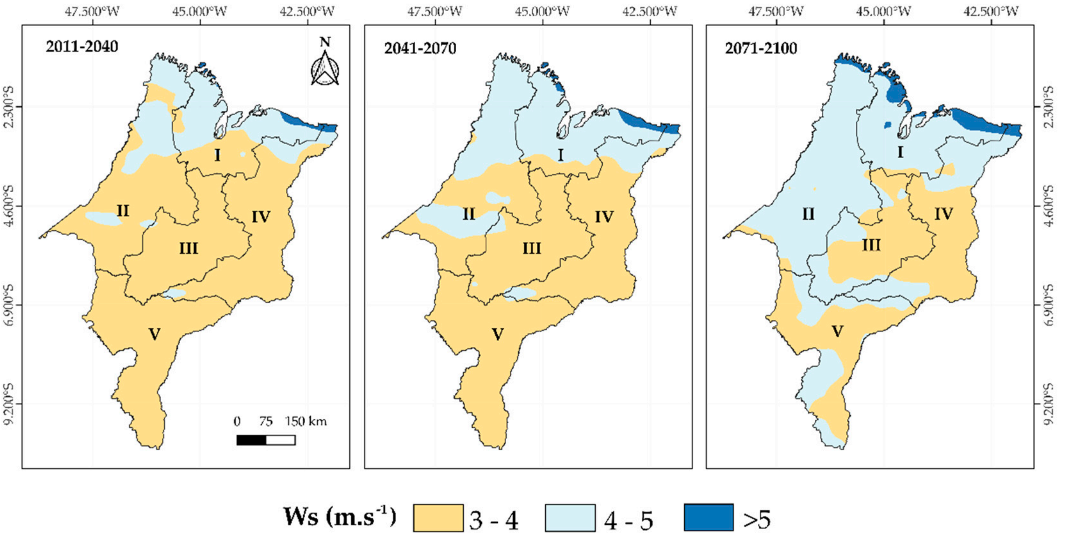


(b)

Figure S8

Kriging maps of Standardized Precipitation Index (SPI) from 2012 to 2023 in the mesoregions of Maranhão: I—North, II—West, III—Central, IV—East, and V—South.

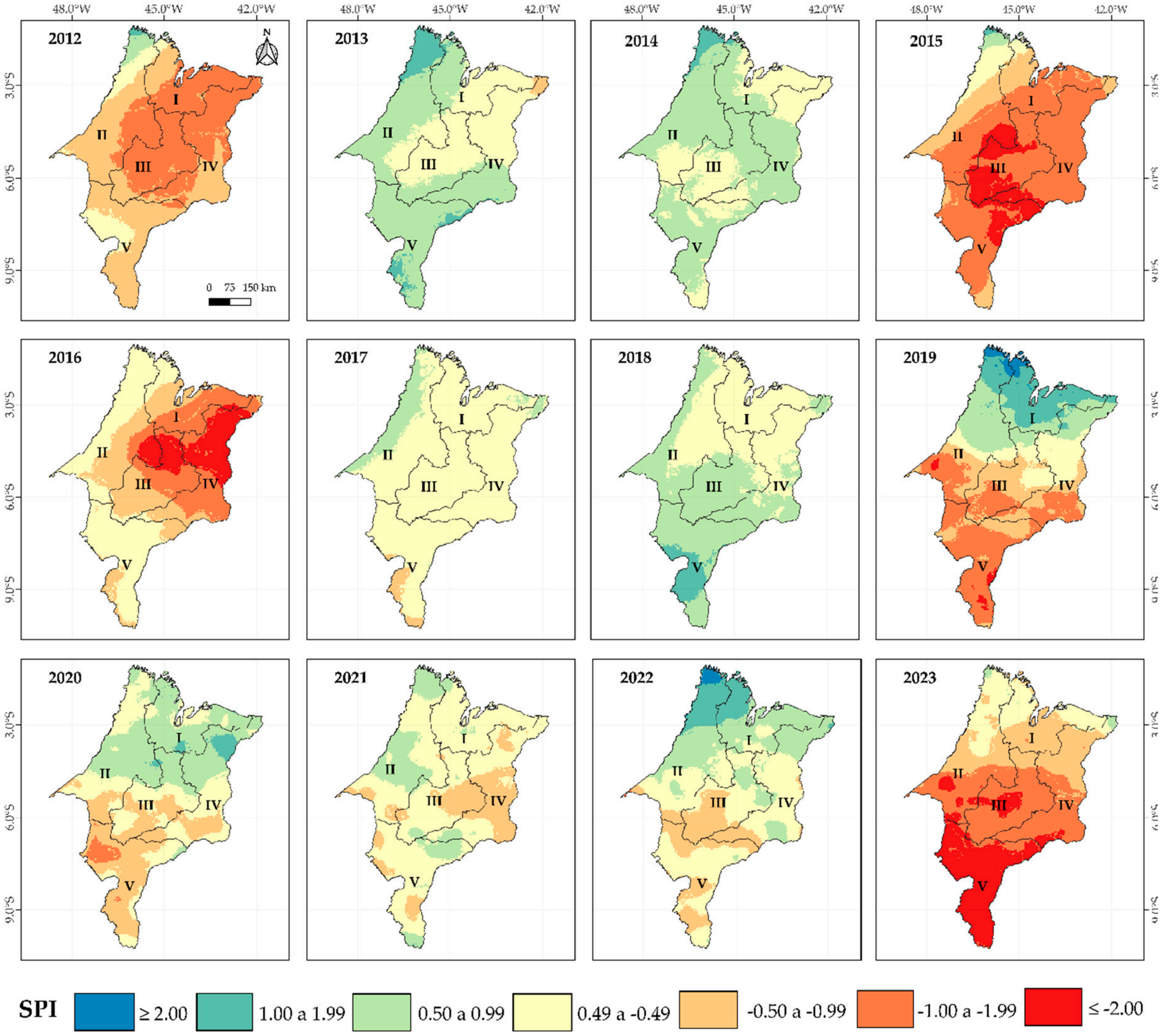

Supplement: Supplementary file 1 [file animals-15-01646-s001.zip › animals-3590865-supplementary.pdf]
